# Simultaneously enhancing organic phosphorescence quantum yields and lifetimes for triphenylphosphine salt doped polymer films†

**DOI:** 10.1039/d4sc00161c

**Published:** 2024-02-26

**Authors:** Jiangang Li, Kuanjian Wei, Jilong Wu, Yuchang Wang, Shujuan Liu, Yun Ma, Qiang Zhao

**Affiliations:** a State Key Laboratory of Organic Electronics and Information Displays & Jiangsu Key Laboratory for Biosensors, Institute of Advanced Materials (IAM) & Institute of Flexible Electronics (Future Technology), Nanjing University of Posts and Telecommunications (NUPT) Nanjing 210023 P. R. China iamyma@njupt.edu.cn iamqzhao@njupt.edu.cn; b College of Electronic and Optical Engineering & College of Flexible Electronics (Future Technology), Jiangsu Province Engineering Research Center for Fabrication and Application of Special Optical Fiber Materials and Devices, Nanjing University of Posts and Telecommunications (NUPT) 9 Wenyuan Road Nanjing 210023 P. R. China

## Abstract

Simultaneously enhancing the quantum yields and luminescence lifetimes of organic persistent room temperature phosphorescence (RTP) molecules is a priority in the organic photonic area, but it remains a formidable challenge. Here, an effective strategy was proposed to improve both quantum efficiencies and emission decay times for phosphorescent triphenylphosphine salts. This approach involves integrating an electron donor unit into a triphenylphosphine salt *via* an alkyl chain. This structure facilitates an intermediate through-space charge transfer excited state, which enhances the intersystem crossing process to boost RTP performance. Moreover, the electron donor moiety contributes additional triplet excitons to the triphenylphosphine salts through triplet-to-triplet energy transfer, substantially increasing the population of triplet excitons. Specifically, compared to butyl(naphthalen-1-yl) diphenylphosphonium bromide (*Φ*_phos._ = 4.9% and *τ* = 255.79 ms), (2-(9*H*-carbazol-9-yl)ethyl)(naphthalen-1-yl)diphenylphosphonium bromide demonstrates a higher phosphorescence quantum yield of 19.6% and an extended emission lifetime of 800.59 ms. This advancement lays the groundwork for developing high-performance organic RTP materials, unlocking new possibilities for advanced photonic applications.

## Abstract 2

The population of triplet excitons of triphenylphosphine salts was increased through intermediate through-space charge transfer and triplet–triplet energy transfer, thereby significantly enhancing their RTP quantum efficiency and lifetime.

## Graphical abstract

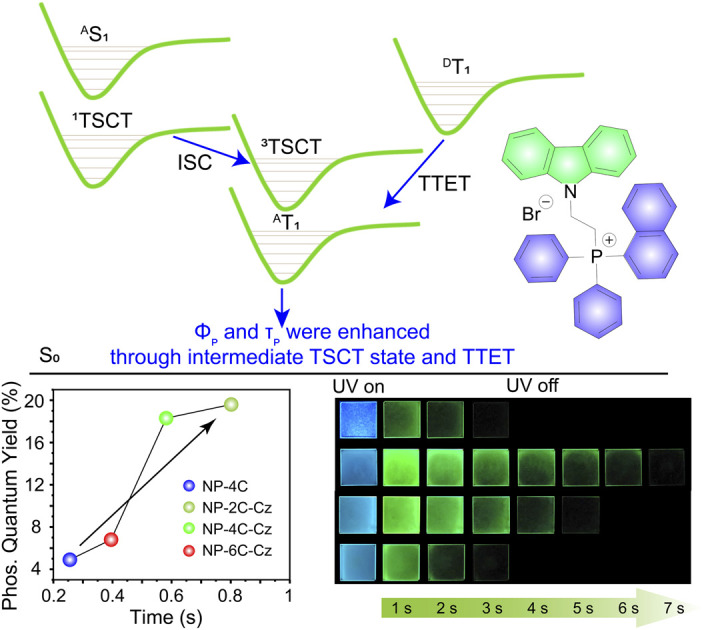

## Introduction

Organic room temperature phosphorescence (RTP) materials with ultralong emission lifetimes have rapidly developed in recent years due to their great potential in diverse areas including bioimaging,^1–4^ X-ray detection,^5–7^ and information encryption.^8–10^ Achieving high quantum yield and long emission decay time is essential for these applications. To this end, different strategies such as crystal engineering,^11–13^ copolymerization,^14–17^ and host–guest doping^18–24^ have been developed to enhance intersystem crossing (ISC) and suppress non-radiative transitions, yielding efficient and long-lived RTP. Notably, the host–guest doping method is noteworthy as it not only stabilizes triplet excitons of organic phosphors within a rigid environment but also allows for persistent RTP in an amorphous state, making it suitable for real-world applications.^25–29^ Particularly, polyvinyl alcohol (PVA) emerges as a promising host matrix due to its water solubility and flexibility, which promote low-cost and eco-friendly processing.^25–27,30–33^ However, a challenge arises as most guest molecules are oil-soluble and tend to undergo phase separation at high doping concentrations in the PVA matrix.^30–33^ Therefore, developing hydrophilic guest molecules that can integrate effectively into a PVA matrix with persistent RTP properties is of crucial importance.

Triphenylphosphine salts have emerged as promising RTP molecules due to their lone pair of electrons on the central phosphorus atom and narrow energy gaps between the lowest singlet (S_1_) and triplet (T_1_) excited states.^14,34–37^ Moreover, these organic salts have good water solubility, which can facilitate uniform dispersion in the PVA matrix. Recently, our group has incorporated triphenylphosphine salts into PVA matrices to create full-color persistent RTP films.^37^ However, the quantum efficiencies and emission lifetimes of these films have not met expectations due to a relatively low intersystem crossing (ISC) rate.

Generally, a key to enhancing both quantum yield and luminescence lifetime is the generation of more triplet excitons.^38,39^ Research has shown that the formation of an intermediate charge transfer (CT) excited state is an effective way to improve the ISC rates of organic phosphors.^40–43^ This can lead to an increase in the production of triplet excitons, potentially enhancing both the quantum efficiency and the emission lifetime of RTP materials. Organic triphenylphosphine salts are typically electron acceptors, but directly conjugating electron donors to them often results in the CT state being the lowest energy state, making it challenging to position its energy level optimally between the S_1_ and T_1_ states.^36,44^ Alternatively, incorporating an electron donor into a triphenylphosphine salt through a non-conjugated alkyl chain not only elevates the energy level but also allows for the precise control of the energy level of CT state.^40,45,46^ This regulation is achieved by adjusting the distance between the donor and acceptor through varying the lengths of the alkyl chains. Also, selecting an electron donor molecule with a suitable triplet energy level can facilitate triplet-to-triplet energy transfer (TTET) from the donor group to the triphenylphosphine salts.^16,47,48^ This process is also important to increase the population of triplet excitons.

**Fig. 1:**
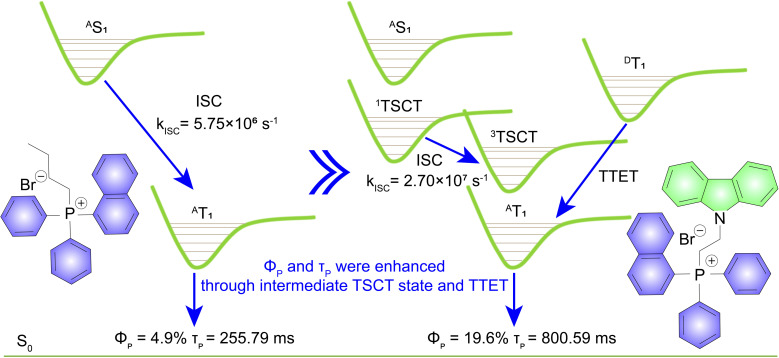
Design strategy for enhancing both quantum yields and lifetimes of triphenylphosphine salts. ^A^S_1_: lowest singlet energy level of the electron acceptor; ^A^T_1_: lowest triplet energy level of the electron acceptor; ^D^T_1_: lowest triplet energy level of the electron donor.

In this work, we have designed and synthesized a series of persistent RTP molecules consisting of triphenylphosphine salts and carbazole units connected by alkyl chains. The selection of carbazole as the electron donor is based on two key reasons. Firstly, it has been demonstrated that carbazole exhibits high quantum yields and long-lived RTP up to several seconds in a PVA matrix.^32,49,50^ Secondly, its lowest triplet energy level is higher than that of triphenylphosphine salts.^45^ The population of triplet excitons in triphenylphosphine salts might be increased due to TTET from the carbazole group (Fig. 1). As expected, the results indicate that by functionalizing with carbazole *via* an alkyl chain, both the phosphorescence quantum yields and lifetimes of the triphenylphosphine salts were significantly enhanced. Therefore, our study demonstrates a promising way for advancing the field of organic phosphorescence.

## Results and discussion

### Synthesis and characterization

**Fig. 2:**
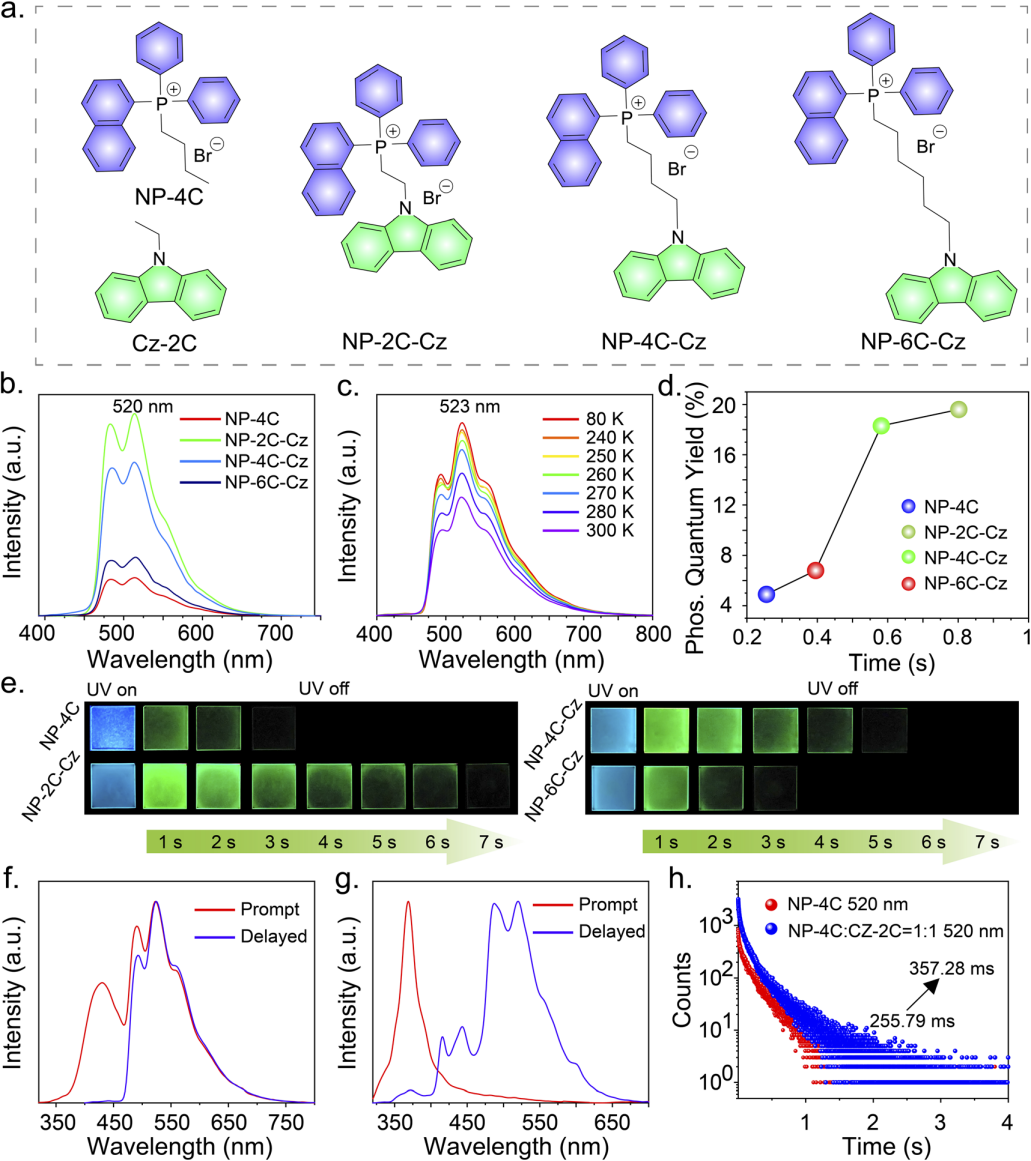
(a) Molecular structures of NP-4C, Cz-2C, NP-2C-Cz, NP-4C-Cz, and NP-6C-Cz. (b) The delayed PL spectra of the compounds in PVA films (Ex = 300 nm). (c) Temperature-dependent delayed PL spectra of NP-2C-Cz in PVA films. (d) Comparative evaluation of phosphorescence quantum efficiencies and lifetimes across different compound-doped PVA films. (e) Afterglow photos of compound-doped PVA films. (f) The PL and delayed PL spectra of NP-2C-Cz in PVA films at 80 K. (g) The PL and delayed PL spectra of a blend of NP-4C and CZ-2C (ratio 1 : 1) in PVA films. (h) Phosphorescence decay curves at 520 nm for PVA films doped with NP-4C and a blend of NP-4C : CZ-2C (ratio 1 : 1).

A series of RTP molecules, including butyl(naphthalen-1-yl)diphenylphosphonium bromide (NP-4C), (2-(9*H*-carbazol-9-yl)ethyl)(naphthalen-1-yl)diphenylphosphonium bromide (NP-2C-Cz), (4-(9*H*-carbazol-9-yl)butyl)(naphthalen-1-yl)diphenylphosphonium bromide (NP-4C-Cz), and (6-(9*H*-carbazol-9-yl)hexyl)(naphthalen-1-yl)diphenylphosphonium bromide (NP-6C-Cz), were designed and synthesized (Fig. 2a). These compounds were prepared by linking carbazole units with triphenylphosphine salts *via* alkyl chains using a two-step method, with the synthesis details provided in the ESI.† The obtained compounds were characterized using ^1^H and ^13^C nuclear magnetic resonance (NMR) spectroscopy, matrix-assisted laser desorption/ionization time-of-flight mass spectrometry (MALDI-TOF-MS), thermogravimetric analysis (TGA) and high-performance liquid chromatography (HPLC) (Fig. S19–S51†).

### Photophysical properties

The photophysical properties of the compounds NP-2C-Cz, NP-4C-Cz, and NP-6C-Cz were investigated in CH_2_Cl_2_ solution at a concentration of 1 × 10^−5^ M (Table S1†). As shown in Fig. S2a,† the UV-visible absorption spectra of these compounds exhibit a composite profile, combining the absorption characteristics of the electron donor and electron acceptor. The maximum absorption peak around 300 nm is attributed to an intramolecular π–π* transition, while the absorption peak in the range of 325–350 nm is associated with an intramolecular n–π* transition. Subsequently, the photoluminescence (PL) spectra of these compounds in CH_2_Cl_2_ solution were studied. Taking NP-2C-Cz as an example, upon excitation at 300 nm, it exhibits dual emission peaks from two distinct excited states. The emission peak at 352 nm is attributable to the combined emission from the carbazole and triphenylphosphorus groups, as shown in Fig. S2b.† This peak remains constant across various solvent polarities, indicating that it is a locally excited (LE) state (Fig. S2c, S3a and b†). Conversely, the emission peak at 478 nm exhibits the characteristic features of a charge-transfer state (CT), varying from 450 nm to 521 nm in the solvents with different polarities (Fig. S2c†). Since the donor (Cz-2C) and acceptor (NP-4C) units are linked by flexible alkyl chains, this transition can be identified as a through-space charge transfer (TSCT) state.^40^ For NP-4C-Cz and NP-6C-Cz, an increase in the alkyl chain length results in enhanced emission intensity from the LE states, while reducing the PL intensity from TSCT excited states. This is further demonstrated by the absorption spectra observed in various polar solvents.

As shown in Fig. S4,† the absorbance of NP-2C-Cz in the range of 350 nm to 450 nm progressively increases with the rising polarity of the solvent. Also, all these emissions originate from singlet excited states due to their nanosecond lifetimes (Fig. S2d and S5†).

### Persistent RTP in PVA

To realize persistent RTP, compounds NP-4C, NP-2C-Cz, NP-4C-Cz, and NP-6C-Cz were doped into a PVA matrix because it can provide rigid networks to stabilize triplet excitons.^25–27^ When excited with 300 nm UV light, the prompt PL spectra of NP-4C, NP-4C-Cz, and NP-6C-Cz doped PVA films displayed PL profiles of the LE state and TSCT state similar to those observed in CH_2_Cl_2_ solution (Fig. S6 and Table S2†). In contrast, the prompt PL spectra of NP-2C-Cz doped PVA films exhibit three distinct peaks: a weak peak at 352 nm corresponding to the ^1^LE state of the carbazole and triphenylphosphine groups, as evidenced by their matching band profile and location; a strong peak at 445 nm, identified as the ^1^TSCT state previously observed in various solvents; another robust peak at 520 nm with a decay lifetime of around 800 ms, attributable to a phosphorescence peak as elucidated in Table S2 and Fig. S6b.†Fig. 2b, c, and e show that all delayed PL peaks of these doped films occur at 520 nm, displaying green persistent RTP. In addition, the delayed PL bands intensify with decreasing temperature from 300 K to 80 K, with the corresponding phosphorescence decay curves at various temperatures showing similar trends (Fig. S7a†). These observations indicate that the 520 nm emission peak stems from the ^3^LE state of the triphenylphosphine unit. The presented data collectively support the concept that TSCT acts as an intermediate excited state bridging the ^1^LE and ^3^LE states of the triphenylphosphine group.

The low-temperature PL spectra provide additional evidence supporting the aforementioned discussed phenomena. Fig. 2f demonstrates that at 80 K, the prompt PL spectra of NP-2C-Cz doped PVA films exhibit emission wavelengths at 431 nm and 523 nm, corresponding to the TSCT excited state and the ^3^LE excited state of the triphenylphosphine unit, respectively. This behavior is also observed in NP-4C-Cz and NP-6C-Cz doped PVA films at 80 K. In addition, the ratio of LE_520nm_/TSCT_445nm_ emissions progressively decreases from 1.7 in NP-2C-Cz to 1.0 in NP-4C-Cz and further to 0.9 in NP-6C-Cz, as indicated in Fig. 2f and S7.† This trend confirms that the TSCT excited state weakens with the increasing distance between the electron donor and acceptor. As anticipated, the phosphorescence quantum efficiency of NP-2C-Cz is four times higher than that of NP-4C, increasing from 4.9% to 19.6%. In the meantime, the emission lifetime at 520 nm enhanced by a factor of three, increasing from 255.79 ms to 800.59 ms under 300 nm excitation, as shown in Fig. 2d and S8.† With the elongation of the alkyl chains, a marked decline is observed in both the phosphorescence quantum efficiencies and lifetimes of the doped PVA films. Specifically, NP-4C-Cz shows a phosphorescence quantum efficiency of 18.3% and a lifetime of 581.80 ms, while NP-6C-Cz exhibits reduced values of 6.8% and 395.26 ms, respectively.

### Mechanism study

To further understand the underlying mechanism of the enhanced RTP performance, a comprehensive investigation of the TTET process was carried out first. As shown in Fig. S9,† the Cz-2C doped PVA film exhibits a blue afterglow at 445 nm with a luminescence lifetime of 1291.32 ms. This property offers the potential to realize TTET from the high-lying T_1_ of the carbazole moiety to the low-lying T_1_ of the triphenylphosphine salt. Next, the photophysical properties of the NP-4C : Cz-2C = 1 : 1 doped PVA film were studied. The steady PL spectrum of this film exhibits mixed PL bands from both NP-4C and Cz-2C, with no emission observed from the charge transfer state, as displayed in Fig. 2g. In addition, the delayed PL spectrum of the NP-4C : Cz-2C = 1 : 1 doped PVA film shows two distinct emission peaks at 445 nm and 520 nm, and these peaks correspond to the triplet excited state of Cz-2C and NP-4C, respectively (Fig. S9a†). The luminescence lifetimes at these wavelengths were measured, revealing a decrease from 1291.32 ms to 1093.36 ms for the donor and an increase from 255.79 ms to 357.28 ms for the acceptor (Fig. 2h and S9b†). Compared to NP-2C-Cz, NP-4C-Cz, and NP-6C-Cz with the alkyl-linked configurations, the emission lifetimes of NP-4C show only a slight increase, which can be attributed to the larger distance between the donor and acceptor. The larger separation in the mixed forms results in less efficient TTET between the donor and acceptor. This observation confirmed the significance of TTET in enhancing the RTP properties of the acceptor. The proximity of the donor and acceptor in alkyl-linked forms facilitates more effective TTET, thereby playing a crucial role in boosting RTP performance.

**Fig. 3:**
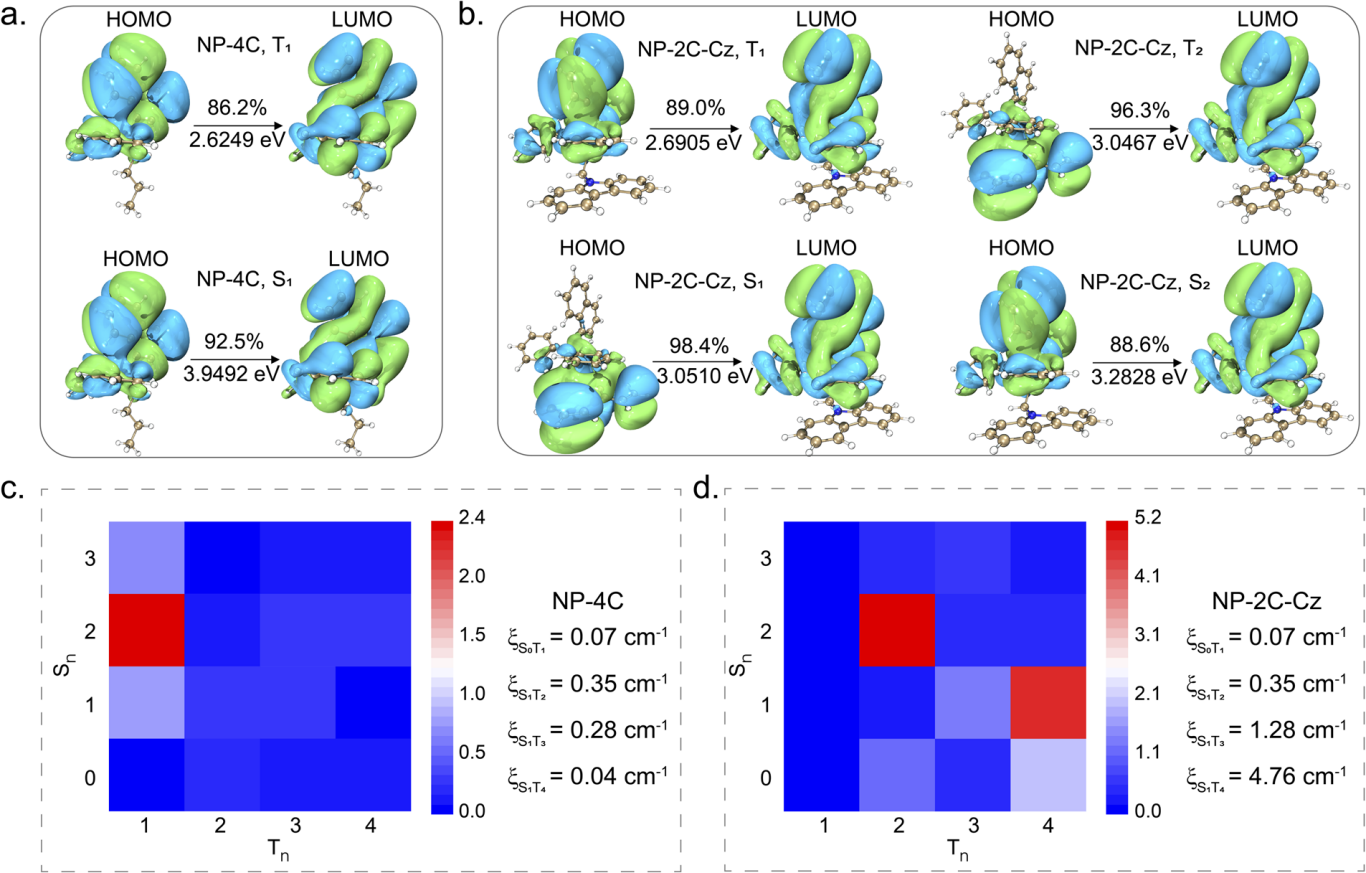
(a and b) Electron transition distributions for NP-4C (S_1_ and T_1_) and NP-2C-Cz (S_1_, S_2_, T_1_, and T_2_) are calculated and illustrated. (c and d) Calculated spin–orbit coupling matrix cm^−1^ for NP-4C and NP-2C-Cz.

Next, the excited state dynamics of NP-4C and NP-2C-Cz were elucidated through theoretical calculations performed at the B3LYP/def2svp level using density functional theory (DFT).^51^ As exhibited in Fig. 3a, for NP-4C, both the highest occupied molecular orbital (HOMO) and the lowest unoccupied molecular orbital (LUMO) are localized on the triphenylphosphine unit, indicating a characteristic LE state. In contrast, for NP-2C-Cz, the HOMO is predominantly located on the carbazole group, while the LUMO is distributed over the triphenylphosphine (Fig. 3b), forming a TSCT state. Single point energy analysis for the S_1_, S_2_, T_1_, T_2,_ T_3_, and T_4_ states of both NP-4C and NP-2C-Cz was also carried out. The analysis revealed that the T_1_ level of NP-4C is at 2.6249 eV, which is closely aligned with the T_1_ level of 2.6905 eV for NP-2C-Cz, both exhibit electron distribution characteristics associated with the triphenylphosphine in an LE state. In NP-2C-Cz, the S_1_ and T_2_ states show the TSCT state, and S_2_ represents the LE state of triphenylphosphine, which indicates that the TSCT state is located between the ^1^LE and ^3^LE of triphenylphosphine. Furthermore, the S_1_ level of NP-2C-Cz at 3.0510 eV displays a remarkably small *Δ*_EST_ (singlet-triplet energy gap) with T_2_ (3.0467 eV), T_3_ (3.1652 eV), and T_4_ (3.2555 eV). The small energy gap is key for efficient ISC, which is crucial in enhancing RTP performance by facilitating a higher population of triplet excitons.

To explore the influence of the TSCT state on spin multiplicity, calculations were carried out to determine the spin–orbit coupling (SOC) for both NP-4C and NP-2C-Cz. As shown in Fig. 3c and d, the results reveal that in the NP-4C geometry, the SOC between the singlet state S_1_ and the triplet states T_3_ or T_4_ is approximately 0.28 cm^−1^ and 0.04 cm^−1^, respectively. In contrast, the SOC between S_1_ and T_3_ or T_4_ in the NP-2C-Cz geometry is significantly increased to 1.28 cm^−1^ and 4.76 cm^−1^, respectively. This is because the formation of the ^3^TSCT state in NP-2C-Cz exhibits a pronounced vibrational coupling with the ^3^LE state. Consequently, the ISC process is enhanced, thereby increasing the population of triplet excitons. This calculational result is in accordance with the experimental data. The intersystem crossing constant (*k*_ISC_) of NP-2C-Cz is calculated to be 2.70 × 10^7^ s^−1^, which is an order of magnitude higher than that of NP-4C at 5.75 × 10^6^ s^−1^ (Table S2†). These data strongly support the effectiveness of incorporating a TSCT state as an intermediate to enhance the exciton dynamics of the ^3^LE state.

### Universality of the design principle

**Fig. 4:**
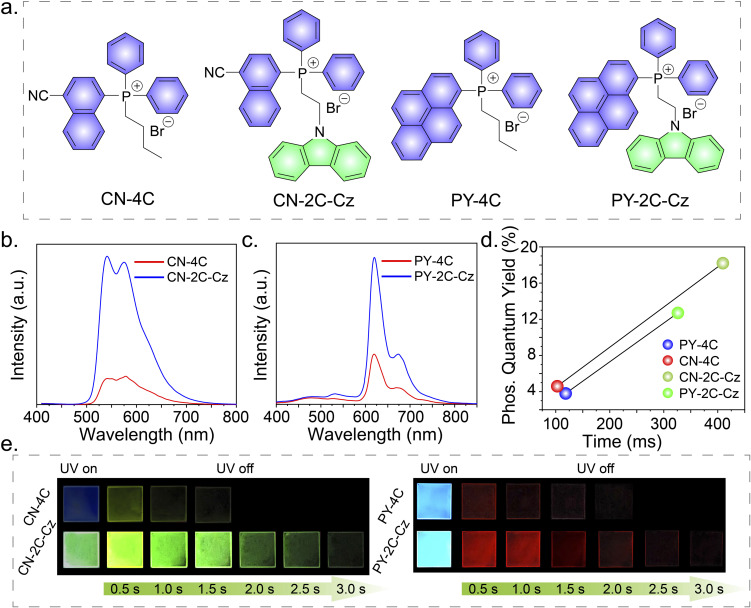
(a) Molecular structures of CN-4C, CN-2C-Cz, PY-4C, and PY-2C-Cz. (b) The delayed PL spectra for PVA films doped with CN-4C and CN-2C-Cz (Ex = 300 nm). (c) The delayed PL spectra for PVA films doped with PY-4C and PY-2C-Cz (Ex = 300 nm). (d) Comparative evaluation of phosphorescence quantum efficiencies and lifetimes for each compound in PVA films. (e) Afterglow photos of compound-doped PVA films.

To demonstrate the universality of this RTP performance improvement strategy, different electron acceptors were synthesized and studied, which include butyl(4-cyanonaphthalen-1-yl) diphenylphosphonium bromide (CN-4C), (2-(9*H*-carbazol-9-yl)ethyl)(4-cyanonaphthalen-1-yl)diphenylphosphonium bromide (CN-2C-Cz), butyldiphenyl(pyren-1-yl)phosphonium bromide (PY-4C), and (2-(9*H*-carbazol-9-yl)ethyl)diphenyl(pyren-1-yl)phosphonium bromide (PY-2C-Cz) (Fig. 4a). The fundamental photophysical properties of these compounds are detailed in Tables S1, S2, and Fig. S10–S14.† The delayed PL spectra of CN-4C and CN-2C-Cz doped PVA films show emission peaks at wavelengths of 540 nm and 576 nm, respectively, as can be seen in Fig. 4b. The delayed PL spectra at 80 K exhibit emission at identical positions (Fig. S13†). Remarkably, the phosphorescence quantum efficiency and lifetime of the CN-2C-Cz doped PVA film increased from 4.6% to 18.2% and from 102.82 ms to 409.44 ms, respectively, compared to the CN-4C doped PVA film (Fig. 4d and e). This enhancement was visually confirmed by a yellow afterglow corresponding to the CIE coordinates (0.45, 0.54) in Fig. S14.† A similar trend was observed in the PY-4C and PY-2C-Cz doped PVA films. The delayed PL spectra of these films show distinct emission peaks at 619 nm and 667 nm, aligning with the emission peaks observed at 80 K (Fig. 4c and S13†).The phosphorescence quantum efficiency and lifetime exhibited a substantial increase from 3.8% to 12.7% and from 118.34 ms to 326.18 ms, respectively (Fig. 4d and e). This resulted in a pronounced enhancement of the visible red afterglow, corresponding to CIE coordinates (0.52, 0.33) in Fig. S14.†

## Applications

**Fig. 5:**
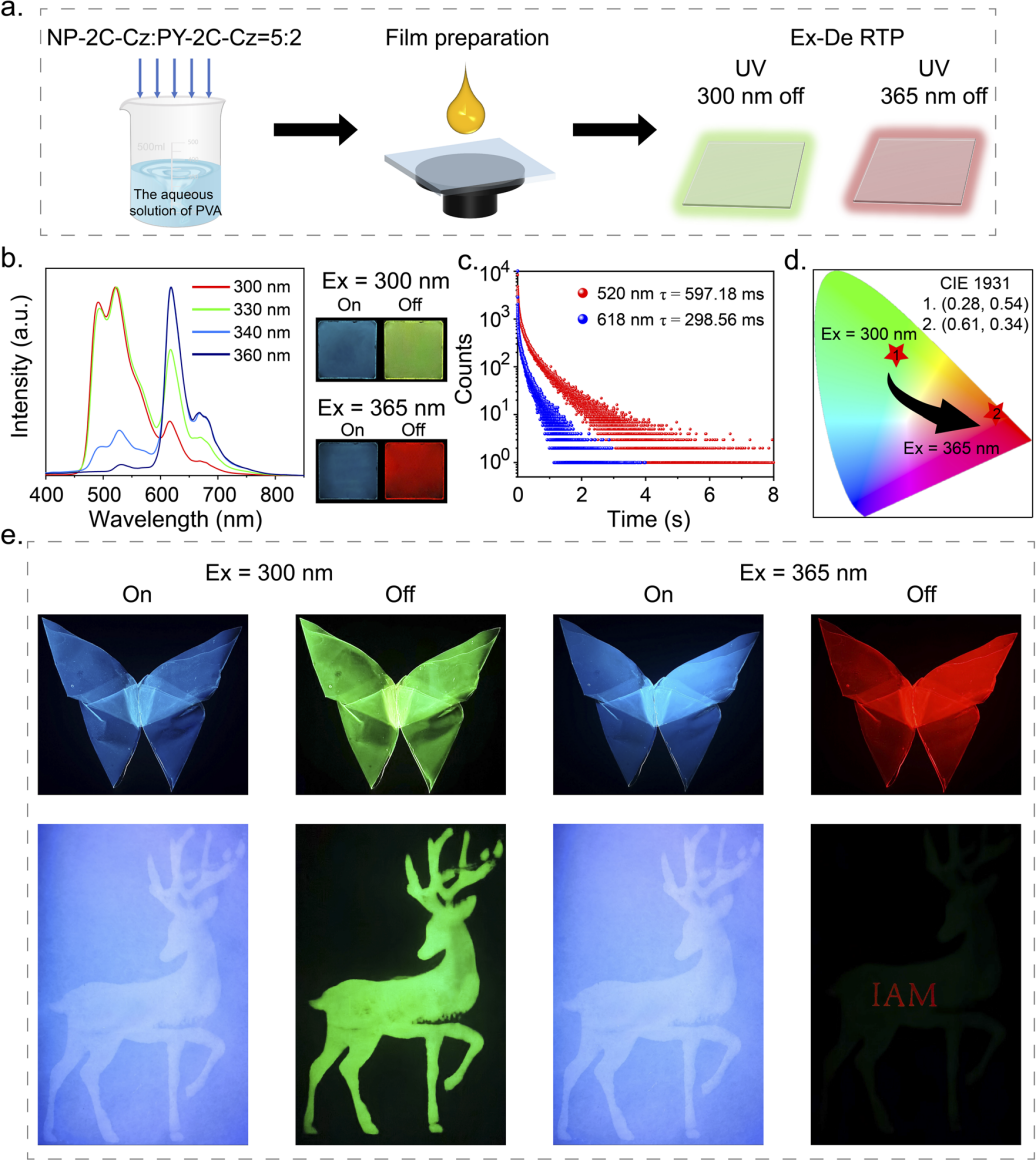
(a) The process for preparing Ex-De persistent RTP films. (b) The delayed PL spectra of PVA films doped with a mixture of NP-2C-Cz and PY-2C-Cz in a 5 : 2 ratio, under varying excitation wavelengths. (c) Phosphorescence decay curves at 520 nm and 618 nm for the NP-2C-Cz and PY-2C-Cz co-doped PVA film, excited at 300 nm and 365 nm, respectively. (d) CIE coordinates for the NP-2C-Cz and PY-2C-Cz co-doped PVA film under 300 nm and 365 nm excitation. (e) Demonstration of the flexibility of the NP-2C-Cz and PY-2C-Cz co-doped PVA film, along with their application in displaying and information encryption.

The excitation spectra of NP-2C-Cz, CN-2C-Cz, and PY-2C-Cz doped PVA films were found to exhibit distinct characteristics. Notably, PY-2C-Cz maintained its excitation band even beyond 350 nm, while NP-2C-Cz doped PVA films could only be excited below 350 nm (Fig. S15a†). In addition, no Ex-De RTP behavior is noticeable in NP-2C-Cz and PY-2C-Cz doped films when exposed to different excitation wavelengths (Fig. S15b and c†). To utilize these differences, NP-2C-Cz and PY-2C-Cz were co-doped into PVA films to create excitation wavelength dependent (Ex-De) persistent RTP films (Fig. 5a). After multiple attempts, the optimal co-doping ratio was determined to be 5 : 2 (NP-2C-Cz : PY-2C-Cz), as indicated in Fig. S16 and S17.† The resulting NP-2C-Cz and PY-2C-Cz co-doped PVA film exhibited a green afterglow (597.18 ms) after stopping 300 nm excitation, and a red afterglow (298.56 ms) upon removal of the 365 nm excitation. This was visually confirmed by the changes in the CIE coordinates from (0.28, 0.54) to (0.61, 0.34). As shown in Fig. 5e, a butterfly with Ex-De properties was created, which showed green persistent RTP after removing 300 nm excitation and transformed into a red afterglow upon removal of the 365 nm excitation. The PVA films doped with a 5 : 2 ratio of NP-2C-Cz and PY-2C-Cz, illustrated in Fig. S18,† were subjected to continuous irradiation with either 300 nm or 365 nm UV light for a duration of 120 min. Remarkably, the emission intensity at both 520 nm and 618 nm remained unchanged during this exposure period. These results demonstrate the outstanding photostability of the co-doped PVA film, making it exceptionally suitable for long-term applications. Further, the NP-2C-Cz PVA solution was used to print a deer pattern, while the NP-2C-Cz and PY-2C-Cz co-doped PVA film was employed to produce the encrypted message “IAM” onto the deer. This resulted in the green deer pattern being visible only under 300 nm excitation, while removing the 365 nm excitation revealed the red encrypted message “IAM”.

## Conclusions

In summary, an effective strategy was proposed to enhance both the RTP quantum efficiency and lifetimes of triphenylphosphine salts in a PVA matrix. Experimental and theoretical results indicate that the introduction of an intermediate TSCT excited state can narrow the energy gap between the singlet and triplet states of triphenylphosphine salts, enhance their SOCs and increase *k*_ISC_ by an order of magnitude. Furthermore, efficient TTET allows the increase of the triplet excitons in the triphenylphosphine salts. The synergistic effect of these two mechanisms significantly increases the population of triplet excitons, effectively bridging the gap between long lifetime and high quantum efficiency in triphenylphosphine salts. The versatility of this approach is demonstrated by the modification of the electron donor group to achieve multi-color persistent RTP. Eventually, the prepared films were successfully applied in anti-counterfeiting and information encryption, demonstrating their practical photonic applications.

## Data availability

All experimental data are available from the corresponding author upon reasonable request.

## Author contributions

Jiangang Li: conceptualization, validation, methodology, investigation, and writing – original draft. Kuanjian Wei, Jilong Wu, and Yuchang Wang: methodology, validation, and investigation. Shujuan Liu and Qiang Zhao: supervision, writing – review & editing, and funding acquisition. Yun Ma: conceptualization, supervision, writing – review & editing, and funding acquisition.

## Conflicts of interest

The authors declare no competing financial interest.

## Supplementary Material

SC-015-D4SC00161C-s001

SC-015-D4SC00161C-s002

## References

[cit1] Chang K., Xiao L., Fan Y., Gu J., Wang Y., Yang J., Chen M., Zhang Y., Li Q., Li Z. (2023). Sci. Adv..

[cit2] Su X., Kong X., Sun K., Liu Q., Pei Y., Hu D., Xu M., Feng W., Li F. (2022). Angew. Chem., Int. Ed..

[cit3] Zhang Y., Li J., Zhao J., Li X., Wang Z., Huang Y., Zhang H., Liu Q., Lei Y., Ding D. (2024). Angew. Chem., Int. Ed..

[cit4] Lei L., Yang F., Meng X., Xu L., Liang P., Ma Y., Dong Z., Wang Y., Zhang X. B., Song G. (2023). J. Am. Chem. Soc..

[cit5] Chen H., Lin M., Zhao C., Zhang D., Zhang Y., Chen F., Chen Y., Fang X., Liao Q., Meng H., Lin M. (2023). Adv. Opt. Mater..

[cit6] Gan N., Zou X., Dong M., Wang Y., Wang X., Lv A., Song Z., Zhang Y., Gong W., Zhao Z., Wang Z., Zhou Z., Ma H., Liu X., Chen Q., Shi H., Yang H., Gu L., An Z., Huang W. (2022). Nat. Commun..

[cit7] Huang J., Su L., Xu C., Ge X., Zhang R., Song J., Pu K. (2023). Nat. Mater..

[cit8] Xie W., Huang W., Li J., He Z., Huang G., Li B. S., Tang B. Z. (2023). Nat. Commun..

[cit9] Lei Y., Dai W., Guan J., Guo S., Ren F., Zhou Y., Shi J., Tong B., Cai Z., Zheng J., Dong Y. (2020). Angew. Chem., Int. Ed..

[cit10] Qiao W., Yao M., Xu J., Peng H., Xia J., Xie X., Li Z. (2023). Angew. Chem., Int. Ed..

[cit11] Abe A., Goushi K., Mamada M., Adachi C. (2023). Adv. Mater..

[cit12] Chong K. C., Chen C., Zhou C., Chen X., Ma D., Bazan G. C., Chi Z., Liu B. (2022). Adv. Mater..

[cit13] Wang J. X., Fang Y. G., Li C. X., Niu L. Y., Fang W. H., Cui G., Yang Q. Z. (2020). Angew. Chem., Int. Ed..

[cit14] Wei J., Liu C., Duan J., Shao A., Li J., Li J., Gu W., Li Z., Liu S., Ma Y., Huang W., Zhao Q. (2023). Nat. Commun..

[cit15] Dou X., Zhu T., Wang Z., Sun W., Lai Y., Sui K., Tan Y., Zhang Y., Yuan W. Z. (2020). Adv. Mater..

[cit16] Wang C., Zhang Y., Wang Z., Zheng Y., Zheng X., Gao L., Zhou Q., Hao J., Pi B., Li Q., Yang C., Li Y., Wang K., Zhao Y. (2022). Adv. Funct. Mater..

[cit17] Li H., Xue X., Cao Y., Cheng H., Luo A., Guo N., Li H., Xie G., Tao Y., Chen R., Huang W. (2023). J. Am. Chem. Soc..

[cit18] Huang G., Deng Z., Pang J., Li J., Ni S., Li J. A., Zhou C., Li H., Xu B., Dang L., Li M. D. (2021). Adv. Opt. Mater..

[cit19] Qiu W., Cai X., Li M., Chen Z., Wang L., Xie W., Liu K., Liu M., Su S. J. (2021). J. Phys. Chem. Lett..

[cit20] Wang Z., Li A., Zhao Z., Zhu T., Zhang Q., Zhang Y., Tan Y., Yuan W. Z. (2022). Adv. Mater..

[cit21] Zhang X., Chong K. C., Xie Z., Liu B. (2023). Angew. Chem., Int. Ed..

[cit22] Ma L., Ma X. (2023). Sci. China: Chem..

[cit23] Sun S., Ma L., Wang J., Ma X., He T. (2022). Natl. Sci. Rev..

[cit24] Ji M., Ma X. (2023). Ind. Chem. Mater..

[cit25] Li D., Yang Y., Yang J., Fang M., Tang B. Z., Li Z. (2022). Nat. Commun..

[cit26] Su Y., Phua S., Li Y., Zhou X., Jana D., Liu G., Lim W. Q., Ong W. K., Yang C., Zhao Y. (2018). Sci. Adv..

[cit27] Tian R., Xu S. M., Xu Q., Lu C. (2020). Sci. Adv..

[cit28] Xiong S., Xiong Y., Wang D., Pan Y., Chen K., Zhao Z., Wang D., Tang B. Z. (2023). Adv. Mater..

[cit29] Wang C., Qu L., Chen X., Zhou Q., Yang Y., Zheng Y., Zheng X., Gao L., Hao J., Zhu L., Pi B., Yang C. (2022). Adv. Mater..

[cit30] Chen K., Xiong Y., Wang D., Pan Y., Zhao Z., Wang D., Tang B. Z. (2023). Adv. Funct. Mater..

[cit31] Gao Y., Ye W., Qiu K., Zheng X., Yan S., Wang Z., An Z., Shi H., Huang W. (2023). Adv. Mater..

[cit32] Zhang Y., Su Y., Wu H., Wang Z., Wang C., Zheng Y., Zheng X., Gao L., Zhou Q., Yang Y., Chen X., Yang C., Zhao Y. (2021). J. Am. Chem. Soc..

[cit33] Zou R., Jin L., Zheng Y., Shao G., Hong W. (2024). Chem. Eng. J..

[cit34] Gao W., Dai X., Hou C., Sun W., Gong Q., Chen H., Ge Y. (2023). Adv. Opt. Mater..

[cit35] Jena S., Behera S. K., Eyyathiyil J., Kitahara M., Imai Y., Thilagar P. (2023). Adv. Opt. Mater..

[cit36] Jena S., Eyyathiyil J., Behera S. K., Kitahara M., Imai Y., Thilagar P. (2022). Chem. Sci..

[cit37] She P., Duan J., Li F., Zhou Y., Qin Y., Wei J., Liu S., Ma Y., Zhao Q. (2023). Sci. China Mater..

[cit38] Tian Y., Yang J., Liu Z., Gao M., Li X., Che W., Fang M., Li Z. (2021). Angew. Chem., Int. Ed..

[cit39] Chen J., Chen X., Cao L., Deng H., Chi Z., Liu B. (2022). Angew. Chem., Int. Ed..

[cit40] Zhang J., Alam P., Zhang S., Shen H., Hu L., Sung H. H. Y., Williams I. D., Sun J., Lam J. W. Y., Zhang H., Tang B. Z. (2022). Nat. Commun..

[cit41] Kongasseri A. A., Garain S., Ansari S. N., Garain B. C., Wagalgave S. M., Singh U., Pati S. K., George S. J. (2023). Chem. Mater..

[cit42] Chen X., Xu C., Wang T., Zhou C., Du J., Wang Z., Xu H., Xie T., Bi G., Jiang J., Zhang X., Demas J. N., Trindle C. O., Luo Y., Zhang G. (2016). Angew. Chem., Int. Ed..

[cit43] Cheng A., Su H., Gu X., Zhang W., Zhang B., Zhou M., Jiang J., Zhang X., Zhang G. (2023). Angew. Chem., Int. Ed..

[cit44] Gao F., Du R., Han C., Zhang J., Wei Y., Lu G., Xu H. (2019). Chem. Sci..

[cit45] Li J. G., Xing G., Wu J., Zhang Y., Wei J., Liu S., Ma Y., Zhao Q. (2024). Laser Photonics Rev..

[cit46] Wang X., Ma H., Gu M., Lin C., Gan N., Xie Z., Wang H., Bian L., Fu L., Cai S., Chi Z., Yao W., An Z., Shi H., Huang W. (2019). Chem. Mater..

[cit47] Zhao W., Cheung T. S., Jiang N., Huang W., Lam J. W. Y., Zhang X., He Z., Tang B. Z. (2019). Nat. Commun..

[cit48] Deng X., Huang J., Wang G., Li J., Li X., Lei C., Zhang K. (2022). Chem. Commun..

[cit49] Hou L., Yu X., Wang G., Huang W., Zhu X., Liu H., Nie L., Zhang W., Qiu J., Xu X., Wang T. (2024). Adv. Opt. Mater..

[cit50] Yang Y., Liang Y., Zheng Y., Li J. A., Wu S., Zhang H., Huang T., Luo S., Liu C., Shi G., Sun F., Chi Z., Xu B. (2022). Angew. Chem., Int. Ed..

[cit51] Lu T. , Molclus program. version1.9.9.9, www.keinsci.comresearchmolclus.html, accessed May12. 2023

