# Supplementary material for: Simultaneously enhancing organic phosphorescence quantum yields and lifetimes for triphenylphosphine salt doped polymer films
Source: Chem Sci. 2024 Feb 26;15(13):4881–9. doi: 10.1039/d4sc00161c (PMC10967014; doi:10.1039/d4sc00161c)
Supplement: SC-015-D4SC00161C-s001 [file SC-015-D4SC00161C-s001.pdf]

Electronic Supplementary Material (ESI) for Chemical Science.
This journal is © The Royal Society of Chemistry 2024

Electronic Supplementary Information

Simultaneously enhancing organic phosphorescence quantum yields and lifetimes for triphenylphosphine salts doped polymer films

Jiangang Li, Kuanjian Wei, Jilong Wu, Yuchang Wang, Shujuan Liu, Yun Ma*, and Qiang Zhao*

1. Synthesis method

Carbazole derivatives were synthesized according to the reported method.[53,54] **9-(2-bromoethyl)-9H-carbazole**: ^{1}H NMR (400 MHz, DMSO-d_6) δ 8.16 (d, J = 7.6 Hz, 2H), 7.67 (d, J = 8.3 Hz, 2H), 7.46 (t, J = 7.1 Hz, 2H), 7.22 (t, J = 7.5 Hz, 2H), 4.86 (t, J = 6.4 Hz, 2H), 3.92 (t, J = 6.3 Hz, 2H). **9-(4-bromobutyl)-9H-carbazole**: ^{1}H NMR (400 MHz, $CDCl_3$-d_1) δ 8.11 (d, J = 7.8 Hz, 2H), 7.52 – 7.44 (m, 2H), 7.40 (d, J = 8.1 Hz, 2H), 7.23 (d, J = 7.9 Hz, 2H), 4.36 (t, J = 6.9 Hz, 2H), 3.38 (t, J = 6.5 Hz, 2H), 2.12 – 2.03 (m, 2H), 1.98 – 1.87 (m, 2H). **9-(6-bromohexyl)-9H-carbazole**: ^{1}H NMR (400 MHz, DMSO-d_6) δ 8.14 (d, *J* = 7.7 Hz, 2H), 7.57 (d, *J* = 6.3 Hz, 2H), 7.44 (t, *J* = 8.3 Hz, 2H), 7.19 (t, *J* = 7.4 Hz, 2H), 4.41 – 4.28 (m, 2H), 3.49 – 3.39 (m, 2H), 1.73 (dd, *J* = 14.7, 7.5 Hz, 4H), 1.40 – 1.21 (m, 4H).

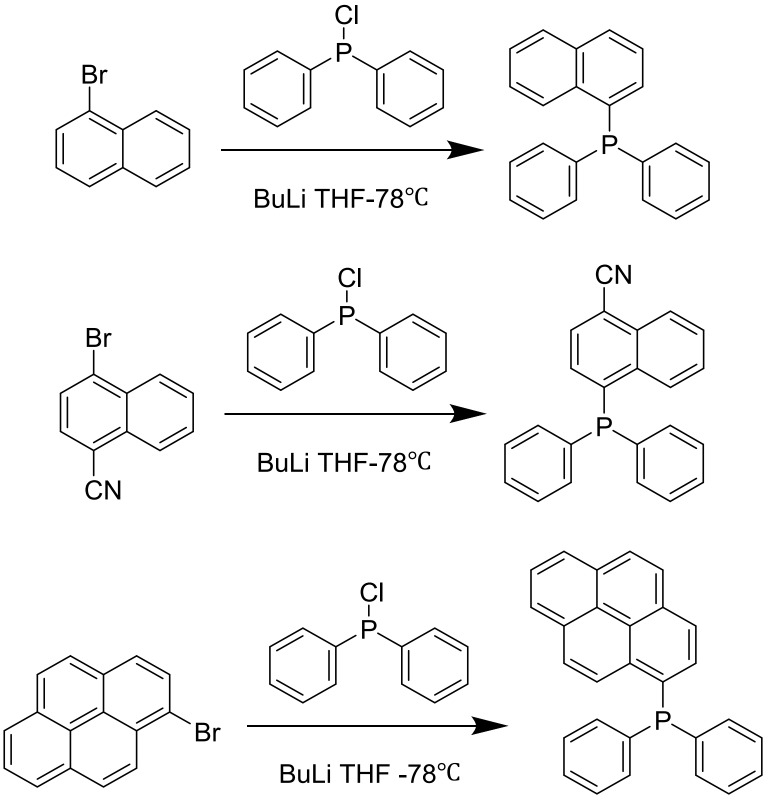

Scheme 1. The synthetic route of **naphthalen-1-yldiphenylphosphane**, **4 (diphenylphosphaneyl)-1-naphthonitrile**, and **diphenyl(pyren-1-yl) phosphane**.

Synthesis of Naphthalen-1-yldiphenylphosphane:

In the N_2 environment, 5.0 g (24.15 mmol, 1.0 eq.) of 1-bromonaphthalene was added to a solution of ultra-dry tetrahydrofuran (THF) with a volume of 60 mL. The reaction mixture was then cooled to an ultra-low temperature (-78 °C) and kept for over 15 minutes. Subsequently, a hexane solution containing n-BuLi at a concentration of 1.6 M (18.11 mL, 28.98 mmol, 1.2 eq.) was slowly added dropwise at a controlled rate while maintaining the reaction temperature at -78 °C and stirring for one hour. After gradually warming the reaction vessel to room temperature, diphenylphosphine chloride (4.8 g, 21.95 mmol, 1.1 eq.) was introduced and stirred for a duration of twelve hours. Upon completion of the reaction, residual n-BuLi was quenched by adding deionized water (20 mL) to the mixed

solution followed by removal of tetrahydrofuran using vacuum distillation via rotary evaporation apparatus. The resulting concentrate was subjected to extraction using a mixture composed of water and methylene chloride as solvents. The organic phase obtained from this process was collected and subsequently dried using anhydrous sodium sulfate. Further purification involved evaporating under reduced pressure to obtain crude product in solid form. Final purification steps were carried out through column chromatography utilizing petroleum ether and dichloromethane as eluent with an overall yield achieved at approximately 90% (Naphthalen-1-yldiphenylphosphane). ^{1}H NMR (400 MHz, DMSO-d_6) δ 8.28 (dd, J = 8.3, 4.3 Hz, 1H), 7.98 (t, J = 7.6 Hz, 2H), 7.56 – 7.39 (m, 9H), 7.28 – 7.21 (m, 4H), 6.98 – 6.90 (m, 1H).

Synthesis of 4-(diphenylphosphaneyl)-1-naphthonitrile:

The synthesis of **4-(diphenylphosphaneyl)-1-naphthonitrile** was accomplished using the same methodology as **Naphthalen-1-yldiphenylphosphane**. Subsequently, the resulting yellow solid underwent further purification via column chromatography employing a petroleum ether and dichloromethane mixture as the eluent, yielding 82.3%. ^{1}H NMR (400 MHz, DMSO-d_6) δ 8.38 (dd, J = 8.3, 4.3 Hz, 1H), 8.19 (d, J = 8.2 Hz, 1H), 8.12 (d, J = 7.5 Hz, 1H), 7.86 – 7.79 (m, 1H), 7.73 – 7.67 (m, 1H), 7.49 – 7.41 (m, 6H), 7.27 (td, J = 7.9, 2.3 Hz, 4H), 7.01 (dd, J = 7.5, 4.0 Hz, 1H).

Synthesis of diphenyl(pyren-1-yl) phosphane:

The synthesis of **diphenyl(pyren-1-yl) phosphane** was accomplished using the same methodology as **Naphthalen-1-yldiphenylphosphane**. The resulting yellow solid was further purified via column chromatography, employing a petroleum ether and dichloromethane mixture as the eluent, yielding 80.1%. ^{1}H NMR (400 MHz, DMSO-d_6) δ 8.65 (dd, J = 9.2, 4.8 Hz, 1H), 8.35 (dd, J = 15.2, 7.8 Hz, 2H), 8.29 – 8.22 (m, 3H), 8.20 – 8.09 (m, 2H), 7.49 (dd, J = 7.9, 4.3 Hz, 1H), 7.42 (d, J = 3.9 Hz, 6H), 7.33 – 7.24 (m, 4H).

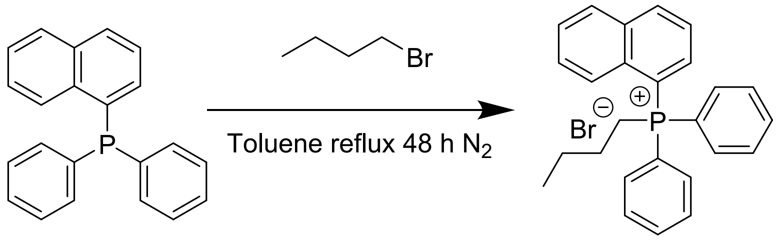

NP-4C

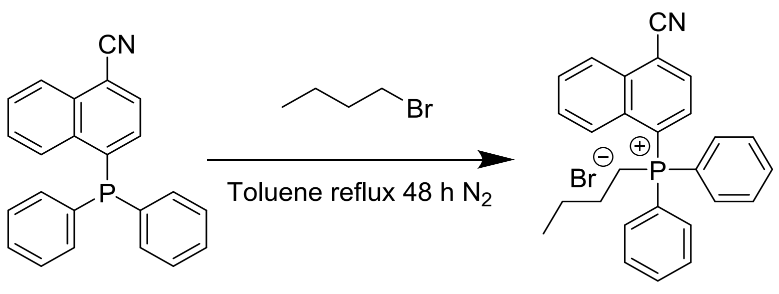

CN-4C

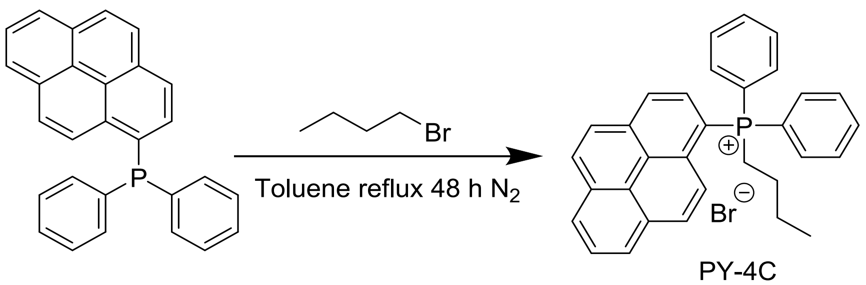

Scheme 2. The synthetic route of **NP-4C**, **CN-4C**, and **PY-4C**.

Synthesis of NP-4C:

A mixture of 500 mg (1.6 mmol, 1.0 eq.) of naphthalen-1-yldiphenylphosphane, 1.09 g (8 mmol, 5 eq.) of 1-bromobutane and 20 ml of ultra-dry toluene was added to a round-bottomed flask with a capacity of 100 ml. The reaction mixture was refluxed under a nitrogen atmosphere for 48 hours before the solvent was removed by rotary evaporation and the remaining residue purified using flash column chromatography with an ethyl acetate:methanol ratio of 100:1 resulting in a white solid product with a yield of 70%. ^{1}H NMR (400 MHz, DMSO-d_6) δ 8.54 (d, J = 8.1 Hz, 1H), 8.24 (d, J = 9.0 Hz, 1H), 7.98 – 7.82 (m, 8H), 7.77 (dd, J = 7.6, 3.6 Hz, 4H), 7.70 (t, J = 8.9 Hz, 2H), 7.55 (t, J = 7.8 Hz, 1H), 3.75 (d, J = 15.4 Hz, 2H), 1.52 – 1.42 (m, 4H), 0.85 (q, J = 7.8 Hz, 3H). ^{13}C NMR (101 MHz, DMSO-d_6) δ 138.20, 138.11, 137.14, 135.31, 135.28, 134.22, 134.13, 133.87, 133.77, 132.28, 132.20, 130.91, 130.79, 129.27, 128.05, 126.37, 126.23, 125.57, 125.51, 119.91, 119.06, 114.40, 113.57, 25.27, 25.23, 23.67, 23.50, 22.60, 22.09, 13.69. ESI-MS: m/z = 396.13 (calc. $C_{26}H_{26}P^{+}$: = 369.47)

Synthesis of CN-4C:

The synthesis of butyl(4-cyanonaphthalen-1-yl) diphenylphosphonium bromide was accomplished using the same methodology as **NP-4C**. The process resulted in a yellow solid product with a yield of 55%. ^{1}H NMR (400 MHz, DMSO-d_6) δ 8.50 (dd, J = 12.7, 7.9 Hz, 1H), 8.37 – 8.26 (m, 1H), 8.18 – 7.68 (m, 14H), 3.82 (d, J = 13.9 Hz, 2H), 1.74 – 1.36 (m, 4H), 0.91 (d, J = 7.0 Hz, 3H). ^{13}C NMR (101 MHz, DMSO-d_6) δ 140.16, 139.95, 136.02, 135.61, 134.50, 134.39, 134.06, 133.96, 132.43, 131.02, 130.90, 128.36, 127.07, 126.71, 123.62, 122.80, 120.61, 119.00, 112.65, 111.72, 105.65, 25.20, 23.70, 23.52, 21.90, 21.66, 13.66. ESI-MS: m/z = 394.40 (calc. $C_{27}H_{25}NP^{+}$: = 394.48)

Synthesis of PY-4C:

The synthesis of butyldiphenyl(pyren-1-yl) phosphonium bromide was accomplished using the same methodology as **NP-4C**. The process resulted in a yellow solid product with a yield of 68%. ^{1}H NMR (400 MHz, DMSO-d_6) δ 8.69 – 8.49 (m, 4H), 8.42 (d, J = 9.0 Hz, 1H), 8.37 – 8.18 (m, 3H), 7.93 (dd, J = 13.0, 8.2 Hz, 7H), 7.77 (td, J = 8.0, 3.4 Hz, 4H), 4.04 – 3.72 (m, 2H), 1.61 – 1.45 (m, 4H), 0.90 – 0.81 (m, 3H). ^{13}C NMR (101 MHz, DMSO-d_6) δ 136.37, 136.34, 135.24, 133.93, 133.83, 133.46, 133.35, 132.18, 131.14, 130.94, 130.82, 129.83, 128.46, 128.20, 127.59, 125.95, 125.81, 124.85, 124.75, 124.25, 124.19, 123.32, 120.55, 119.71, 109.55, 108.70, 25.35, 23.73, 23.56, 23.09, 22.59, 13.71. ESI-MS: m/z = 443.37 (calc. $C_{32}H_{28}P^{+}$: = 443.55)

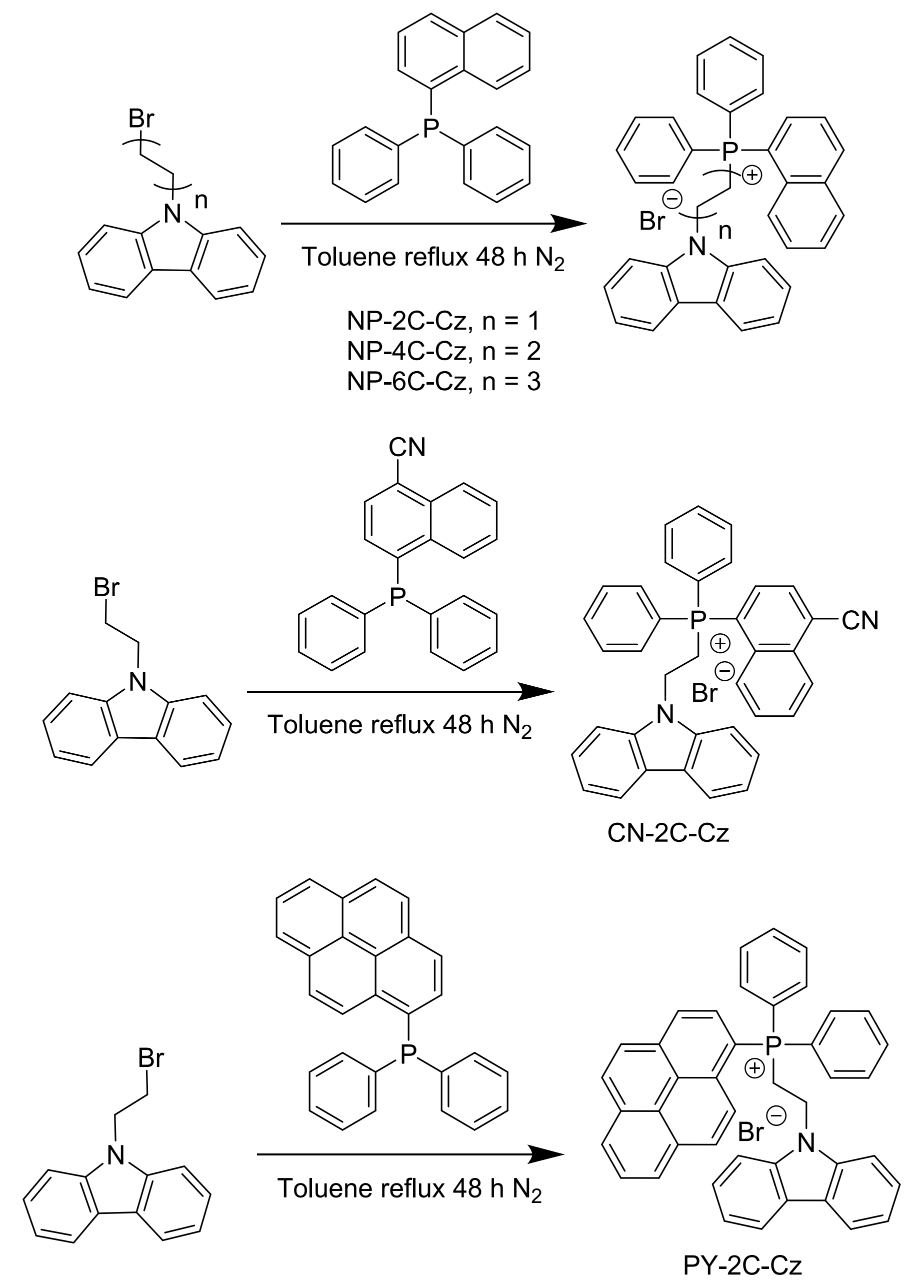

Scheme 3. The synthetic route of **NP-2C-Cz**, **NP-4C-Cz**, **NP-6C-Cz**, **CN-2C-Cz**, and **PY-2C-Cz**.

Synthesis of NP-2C-Cz:

500 mg (1.8 mmol, 1.0 eq.) of 9-(2-bromoethyl)-9H-carbazole, along with 684 mg (2.0 mmol, 1.1 eq.) of naphthalen-1-yldiphenylphosphane and 20 ml of ultra-dry toluene, were introduced into a round-bottomed bottle with a capacity of 100 ml. The reaction mixture was subjected to reflux under a nitrogen atmosphere for a duration of 48 hours. Subsequently, the solvent was eliminated through rotary evaporation and the remaining residue underwent purification via flash column chromatography using a mixture consisting of ethyl acetate and methanol in a ratio of 80:1. This process yielded a white solid product at an efficiency rate of 60%. ^{1}H NMR (400 MHz, DMSO-d_6) δ 8.56 (d, J = 8.3 Hz, 1H), 8.25 (d, J = 8.2 Hz, 1H), 8.11 (d, J = 7.6 Hz, 2H), 8.08 – 7.82 (m, 9H), 7.82 – 7.67 (m, 6H), 7.52 (t, J = 7.8 Hz, 1H), 7.36 (t, J = 7.7 Hz, 2H), 7.23 – 7.12 (m, 4H), 4.71 (q, J = 8.6 Hz, 2H),

4.36 (p, *J* = 8.0 Hz, 2H). ^{13}C NMR (101 MHz, DMSO-d_6) δ 139.48, 138.77, 138.66, 137.43, 137.39, 135.53, 135.49, 134.13, 134.02, 132.16, 132.08, 130.93, 130.80, 129.35, 128.10, 126.36, 126.18, 125.58, 125.52, 122.93, 120.80, 119.90, 119.33, 118.48, 113.37, 112.53, 109.69, 37.05, 23.38, 22.91. ESI-MS: m/z = 506.56 (calc. $C_{36}H_{29}NP^+$: = 506.61)

Synthesis of NP-4C-Cz:

The synthesis of **NP-4C-Cz** was accomplished using the same methodology as **NP-2C-Cz**. The final product obtained was a white solid, with a yield of 65%. ^{1}H NMR (400 MHz, DMSO-d_6) δ 8.51 (d, *J* = 8.1 Hz, 1H), 8.20 (d, *J* = 8.3 Hz, 1H), 8.11 (d, *J* = 7.7 Hz, 2H), 7.93 – 7.79 (m, 4H), 7.78 – 7.62 (m, 9H), 7.54 (dd, *J* = 13.9, 8.3 Hz, 3H), 7.45 (t, *J* = 7.6 Hz, 1H), 7.39 (t, *J* = 7.6 Hz, 2H), 7.18 (t, *J* = 7.5 Hz, 2H), 4.40 (t, *J* = 6.9 Hz, 2H), 3.89 – 3.66 (m, 2H), 1.96 (p, *J* = 7.1 Hz, 2H), 1.60 (t, *J* = 7.9 Hz, 2H). ^{13}C NMR (101 MHz, DMSO-d_6) δ 140.27, 138.07, 137.97, 137.19, 137.15, 135.28, 135.25, 134.17, 134.08, 133.74, 133.64, 132.20, 132.11, 130.84, 130.71, 129.15, 127.97, 126.24, 126.06, 125.44, 125.38, 122.45, 120.72, 119.58, 119.17, 118.74, 114.16, 113.33, 109.70, 41.81, 29.86, 29.70, 22.55, 22.05, 21.07, 21.04. ESI-MS: m/z = 534.56 (calc. $C_{38}H_{33}NP^+$: = 534.66)

Synthesis of NP-6C-Cz:

The synthesis of **NP-6C-Cz** was accomplished using the same methodology as **NP-2C-Cz**. The final product obtained was a white solid, with a yield of 80%. ^{1}H NMR (400 MHz, DMSO-d_6) δ 8.52 (d, *J* = 7.8 Hz, 1H), 8.21 (d, *J* = 8.4 Hz, 1H), 8.14 (d, *J* = 7.7 Hz, 2H), 7.91 – 7.79 (m, 8H), 7.73 (td, *J* = 7.8, 3.7 Hz, 4H), 7.69 – 7.62 (m, 2H), 7.53 (d, *J* = 8.3 Hz, 3H), 7.42 (t, *J* = 7.6 Hz, 2H), 7.18 (t, *J* = 7.5 Hz, 2H), 4.31 (t, *J* = 7.0 Hz, 2H), 3.69 (d, *J* = 14.4 Hz, 2H), 1.64 (p, *J* = 7.3 Hz, 2H), 1.46 (d, *J* = 7.2 Hz, 4H), 1.26 (t, *J* = 6.7 Hz, 2H). ^{13}C NMR (101 MHz, DMSO-d_6) δ 140.37, 138.17, 138.08, 137.17, 137.13, 135.31, 135.28, 134.19, 134.10, 133.83, 133.73, 132.26, 132.17, 130.90, 130.77, 129.26, 128.03, 126.33, 126.19, 126.12, 125.52, 125.45, 122.46, 120.75, 119.83, 119.11, 118.99, 114.33, 113.50, 109.64, 42.43, 30.02, 29.85, 28.47, 25.83, 23.01, 22.96, 22.71, 22.22. ESI-MS: m/z = 562.52 (calc. $C_{40}H_{37}NP^+$: = 562.72)

Synthesis of CN-2C-Cz:

The synthesis of **CN-2C-Cz** was accomplished using the same methodology as **NP-2C-Cz**. The final product obtained was a yellow solid, with a yield of 38%. ^{1}H NMR (400 MHz, DMSO-d_6) δ 8.25 – 8.16 (m, 2H), 8.03 – 7.91 (m, 8H), 7.86 – 7.75 (m, 6H), 7.66 (d, *J* = 8.7 Hz, 1H), 7.45 – 7.28 (m, 4H), 7.20 (d, *J* = 8.3 Hz, 2H), 7.15 (t, *J* = 7.9 Hz, 2H), 4.89 (p, *J* = 6.7 Hz, 2H), 4.56 (dt, *J* = 13.3, 7.0 Hz, 2H). ^{13}C NMR (101 MHz, DMSO-d_6) δ 139.32, 137.44, 137.34, 135.79, 135.75, 134.18, 134.07, 132.42, 132.28, 131.75, 131.65, 131.30, 131.22, 131.05, 130.92, 130.43, 129.83, 126.60, 126.46, 126.39, 126.11, 122.68, 120.62, 119.81, 119.66, 119.04, 118.86, 118.19, 116.80, 116.76, 109.81, 37.15, 23.17, 22.70. ESI-MS: m/z = 531.40 (calc. $C_{37}H_{28}N_2P^+$: = 531.62)

Synthesis of PY-2C-Cz:

The synthesis of **PY-2C-Cz** was accomplished using the same methodology as **NP-2C-Cz**. The final product obtained was a yellow solid, with a yield of 58%. ^{1}H NMR (400 MHz, DMSO-d_6) δ 8.64 – 8.55 (m, 3H), 8.51 (d, *J* = 7.3 Hz, 1H), 8.42 (d, *J* = 8.9 Hz, 1H), 8.35 – 8.26 (m, 2H), 8.20 (d, *J* = 9.3 Hz, 1H), 8.08 – 7.90 (m, 9H), 7.79 (td, *J* = 7.8, 3.5 Hz, 4H),

7.26 (t, J = 7.6 Hz, 2H), 7.19 (d, J = 8.1 Hz, 2H), 7.11 (t, J = 7.4 Hz, 2H), 4.80 (t, J = 7.4 Hz, 2H), 4.51 (p, J = 7.6 Hz, 2H). ^{13}C NMR (101 MHz, DMSO-d_6) δ 139.46, 136.56, 135.47, 134.18, 134.08, 133.83, 133.72, 133.63, 132.24, 131.15, 130.96, 130.84, 130.01, 129.82, 128.64, 128.53, 128.15, 127.59, 126.06, 125.87, 125.73, 124.82, 124.71, 124.14, 124.06, 123.28, 122.83, 120.59, 120.08, 119.78, 119.23, 109.66, 108.35, 107.50, 37.16, 23.88, 23.41. ESI-MS: m/z = 580.49 (calc. $C_{42}H_{31}NP^{+}$: = 580.69)

2. Experimental Methods

Characterization methods:

The UV-Vis absorption spectra were measured utilizing a Shimadzu UV-2600 UV-vis spectrophotometer. The prompt and delayed PL spectra were obtained using a Hitachi F-4700 instrument. Luminous lifetime was determined on an Edinburgh Instruments FLS980 fluorescence spectrophotometer equipped with a microsecond flash-lamp (uF900) and EPLED-280. An 8-watt halogen tube emitting UV light with λ_{max} wavelengths of 300 nm and 365 nm was employed as the light source for photography. The ^{1}H NMR (400 MHz) and ^{13}C NMR (100 MHz) spectra were acquired using a Bruker ACF400 spectrometer at 298 K, employing deuterated solvents (DMSO-d_6). Mass spectra were recorded on a Bruker autoflex MALDI-TOF MS instrument.

Preparation of the PVA doped film:

First, PVA (1 g) was dissolved in deionized water (20 mL) and **NP-2C-Cz** (10 mg) was dissolved in ethanol (1 mL). Then, 1mL of PVA aqueous solution and 100 μL of **NP-2C-Cz** ethanol solution were put into 3mL centrifuge tube respectively, and ultrasonic examination was conducted until clear and transparent. The doped aqueous solution is then coated on the quartz sheet, allowed to evaporate, and dry overnight, and then dried at 75 ° C for 3 hours. Other PVA doped films were prepared by the same method.

Theoretical calculation:

The DFT calculations were performed using Gaussian 16 and ORCA 5.0.2 programs, while Multiwfn and Visual Molecular Dynamics (VMD) software were utilized for visualizing the electron orbital distribution. The ground state (S_0) geometry of **NP-4C** and **NP-2C-Cz** molecules was optimized at the B3LYP/def2svp level, followed by vibration analysis to ensure absence of negative frequencies. Hole electron analysis was conducted on the optimized molecular configuration at the same level, and VMD 1.9.4a48 software was employed to plot the distribution of hole electron orbitals. Finally, spin orbit mean field (SOMF) method was used to calculate the coupling matrix elements between singlet and triplet states.[51,52] The molecular model's Cartesian coordinates are as follows：

NP-4C:

C	-2.41799	0.56565	-3.20008
C	-1.18531	0.38204	-2.53545
C	-1.13521	0.15923	-1.16342
C	-2.35112	0.09876	-0.39109
C	-3.59721	0.29547	-1.08296
C	-3.59503	0.52858	-2.48547
P	0.48501	-0.00895	-0.36505

C	0.69161	1.29382	0.88604
C	0.67915	-1.66018	0.35263
C	1.78083	-1.95188	1.1815
C	1.95816	-3.24708	1.66995
C	1.04752	-4.25541	1.33512
C	-0.04411	-3.96934	0.50993
C	-0.23242	-2.67655	0.0164
C	0.77611	1.02745	2.26228
C	0.91665	2.08303	3.16679
C	0.96892	3.40171	2.70676
C	0.87392	3.67102	1.33679
C	0.73293	2.62327	0.42646
C	-2.39884	-0.15538	1.00745
C	-3.60074	-0.19882	1.68513
C	-4.82363	0.00719	1.00332
C	-4.81798	0.24683	-0.35362
C	1.79461	0.23995	-1.62476
C	3.23064	0.19639	-1.08598
C	4.2547	0.55417	-2.17091
C	5.69436	0.49071	-1.66158
H	-2.42586	0.73923	-4.27782
H	-0.27556	0.42309	-3.13448
H	2.49838	-1.17553	1.45254
H	2.81164	-3.46931	2.31426
H	1.18993	-5.26778	1.72023
H	-0.75631	-4.75504	0.24869
H	-1.0911	-2.46239	-0.6226
H	0.72434	0.00466	2.63862
H	0.98206	1.87145	4.2363
H	1.07851	4.22401	3.41752
H	0.90512	4.70138	0.97585
H	0.64325	2.85142	-0.63876
H	-1.48046	-0.32852	1.56519
H	-3.60724	-0.39639	2.75952
H	-5.76626	-0.02867	1.55361
H	-5.75579	0.40106	-0.8929
H	1.64184	-0.53478	-2.39314
H	1.59241	1.21552	-2.09564
H	3.33548	0.89486	-0.23783
H	3.45247	-0.81097	-0.69877
H	4.12887	-0.13141	-3.0277
H	4.0368	1.56682	-2.55491
H	6.40881	0.75479	-2.45571
H	5.85306	1.18891	-0.8233

H	5.9487	-0.52159	-1.30679
H	-4.55144	0.67565	-2.99367

NP-2C-Cz:

C	-2.50728	-3.08432	-2.66985
C	-1.58519	-2.65287	-1.7076
C	-1.84784	-1.55869	-0.90877
C	-3.08059	-0.84743	-1.04146
C	-4.01088	-1.30099	-2.03066
C	-3.6908	-2.41756	-2.82916
P	-0.61052	-1.01515	0.29027
C	-0.17746	0.69669	-0.06303
C	-1.25385	-1.26179	1.9546
C	-0.59672	-0.72996	3.0649
C	-1.07063	-0.99534	4.33909
C	-2.19439	-1.791	4.51374
C	-2.8457	-2.32504	3.41066
C	-2.38125	-2.06514	2.13179
C	-0.33266	1.72667	0.8628
C	-0.02526	3.02553	0.49772
C	0.42517	3.30216	-0.78476
C	0.56819	2.27854	-1.70987
C	0.26836	0.9752	-1.35546
C	-3.4471	0.25678	-0.24601
C	-4.65096	0.8883	-0.42205
C	-5.5558	0.44883	-1.40057
C	-5.23907	-0.62514	-2.18685
C	0.84988	-2.09524	0.08138
C	2.10442	-1.77432	0.89834
H	-2.27292	-3.9429	-3.28211
H	-0.66274	-3.20332	-1.61036
H	0.27364	-0.10542	2.93995
H	-0.56249	-0.5759	5.19602
H	-2.56282	-1.99459	5.50949
H	-3.72149	-2.94393	3.54417
H	-2.89725	-2.47889	1.27794
H	-0.70244	1.52848	1.85621
H	-0.13225	3.82068	1.22099
H	0.66559	4.31855	-1.06388
H	0.92442	2.48877	-2.70758
H	0.38231	0.18579	-2.08566
H	-2.77886	0.62273	0.5163
H	-4.90559	1.73427	0.2011
H	-6.50038	0.95845	-1.52776

H	-5.93115	-0.976	-2.94052
H	0.51404	-3.10106	0.33169
H	1.09501	-2.08247	-0.9766
H	1.87495	-1.73791	1.95829
H	2.78744	-2.61557	0.76451
H	-4.40459	-2.7452	-3.57348
C	4.14445	0.82725	-3.1252
C	3.79457	-0.5224	-3.05419
C	3.32613	-1.08521	-1.87513
C	3.20912	-0.26	-0.76391
C	3.58038	1.10405	-0.81559
C	4.04889	1.64168	-2.00889
H	4.51366	1.23235	-4.05669
H	3.90562	-1.14815	-3.92879
H	3.10893	-2.14377	-1.83194
H	4.34262	2.68133	-2.06408
C	2.86392	0.59542	1.30264
C	2.61394	0.77996	2.6548
C	2.79174	2.0517	3.18224
C	3.22858	3.11208	2.38744
C	3.52675	2.91293	1.04955
C	3.35493	1.64969	0.4961
H	2.35188	-0.0418	3.3062
H	2.61374	2.21418	4.23639
H	3.36674	4.08825	2.83027
H	3.90094	3.72893	0.44603
N	2.75509	-0.55003	0.519

Fluorescence and phosphorescence QY calculation：

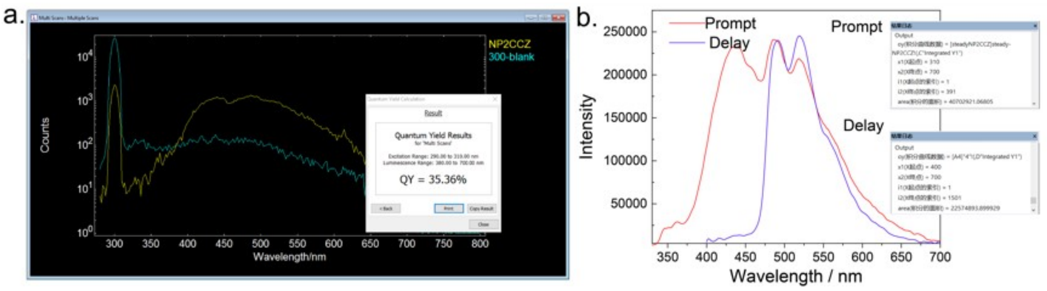

Fig. S1. a. The PL spectra of **NP-2C-Cz** doped PVA film and blank reference PL spectra at 300 nm excitation, b The PL spectra and delay PL of **NP-2C-Cz** doped PVA film.

The fluorescence and phosphorescence quantum yields were separately calculated according to previous literature.[55] The delayed emission spectrum provided the phosphorescence bands. By analyzing the structure of these bands, the fluorescence and phosphorescence emission bands could be distinguished in steady state emission spectra. The ratio between fluorescence and phosphorescence quantum yields was determined by

calculating the areas under their respective bands. Therefore, the fluorescence and phosphorescence quantum yields were obtained based on their total luminescence quantum yields and the ratio between their relative quantum yields.

Photoluminescence quantum efficiency was determined using an Edinburgh FLS980 spectrometer with a 142 mm diameter integrating sphere under ambient conditions. The fluorescence and phosphorescence quantum efficiencies (Φ_F and Φ_P) were calculated using the following formulas:

$$\Phi_P = \Phi_E \times \frac{A_P}{A_E} \quad (1)$$

$$\Phi_F = \Phi_E - \Phi_P \quad (2)$$

where Φ_E represents the measured total emission quantum efficiency, A_P refers to the integral area of the phosphorescent component in photoluminescence spectra, and A_E refers to the integral area of all components in photoluminescence spectra except for phosphorescent component.

3. Photophysical property

Table S1 Summary of photophysics of compounds in CH_2Cl_2 1×10^{-5} M.

Compounds CH_2Cl_2 1×10^{-5} M 298 K	Absorption λ_{max}/nm (ε/dm^3 mol^{-1} cm^{-1})	Emission λ_{max}/nm (τ_o/ns)
**NP-4C**	301 (10582)	356 (2.01)
**NP-2C-Cz**	291 (22050), 325 (6048), 338 (6105)	352 (2.32), 478 (10.05)
**NP-4C-Cz**	293 (17388), 323 (6048), 344 (3409)	353 (2.70), 477 (14.17)
**NP-6C-Cz**	294 (12974), 322 (4209), 345 (2554)	352 (3.01), 508 (13.75)
**CN-4C**	323 (6082)	381 (3.09)
**CN-2C-Cz**	293 (20479), 323 (14611), 336 (13805)	381 (3.33), 518 (12.53)
**PY-4C**	284 (25301), 356 (19024), 366 (18896), 384 (14949)	391 (4.19)
**PY-2C-Cz**	284 (40696), 357 (29363), 366 (29491), 385 (22831)	393 (4.38), 497 (14.02)
**Cz-2C**	295 (23062), 331 (5153), 346 (5783)	355 (1.55), 370 (1.57)

Table S2 Summary of photophysics of compounds in PVA doped films (Wt = 2%).

**Compounds doped PVA 2 wt% 298 K**	**λ_{max}/nm (τ_F/ns)**	**Φ_F/%**	**λ_{max}/nm (τ_P/s)**	**Φ_P/%**	**k_{nr}/s^{-1}**	**k_{ISC}/s^{-1}**
**NP-4C**	352 (8.52)	16.9	520 (0.26)	4.9	3.66	5.75×10^{6}
**NP-2C-Cz**	445 (7.25)	15.8	520 (0.80)	19.6	1.01	2.70×10^{7}
**NP-4C-Cz**	454 (8.69)	19.5	520 (0.58)	18.3	1.41	2.11×10^{7}
**NP-6C-Cz**	452 (8.56)	28.8	520 (0.40)	6.8	2.33	7.94×10^{6}
**CN-4C**	374 (6.75)	20.2	540 (0.10)	4.6	9.54	6.81×10^{6}
**CN-2C-Cz**	515 (8.13)	10.5	540 (0.41)	18.2	2.00	2.24×10^{7}
**PY-4C**	415 (13.24)	15.3	619 (0.12)	3.8	8.02	2.87×10^{6}
**PY-2C-Cz**	492 (7.70)	22.3	619 (0.33)	12.7	2.65	1.65×10^{7}

τ_F: lifetime of fluorescence; Φ_F: absolute quantum yield of fluorescence; τ_P: lifetime of phosphorescence; Φ_p: absolute quantum yield of phosphorescence; k_{nr}: rate constant of non-radiative decay of T_1; k_{ISC}: rate constant of intersystem crossing (ISC) from singlet to triplet states. $k_{nr} = (1-\Phi_p)/\tau_P$; $k_{ISC} = \Phi_p/\tau_F$.

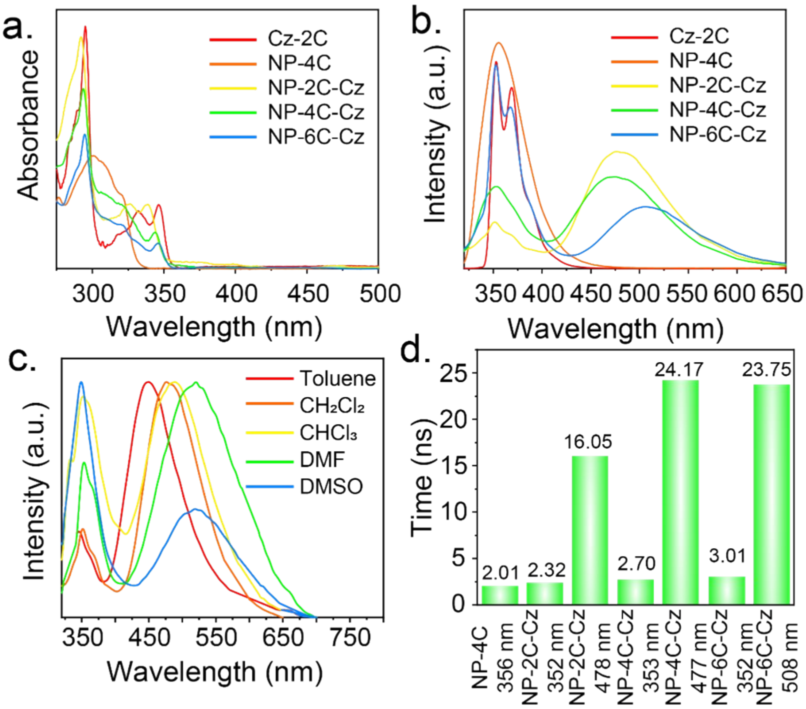

Fig. S2. In CH_2Cl_2 1×10^{-5} M a. The UV-visible absorption spectra of **Cz-2C**, **NP-4C**, **NP-2C-Cz**, **NP-4C-Cz**, and **NP-6C-Cz**. b. The prompt PL spectra (Ex=300 nm) c. The prompt PL spectra of **NP-2C-Cz** in various solution (Ex=300 nm) d. Fluorescence lifetime in solution (Ex = 280 nm)

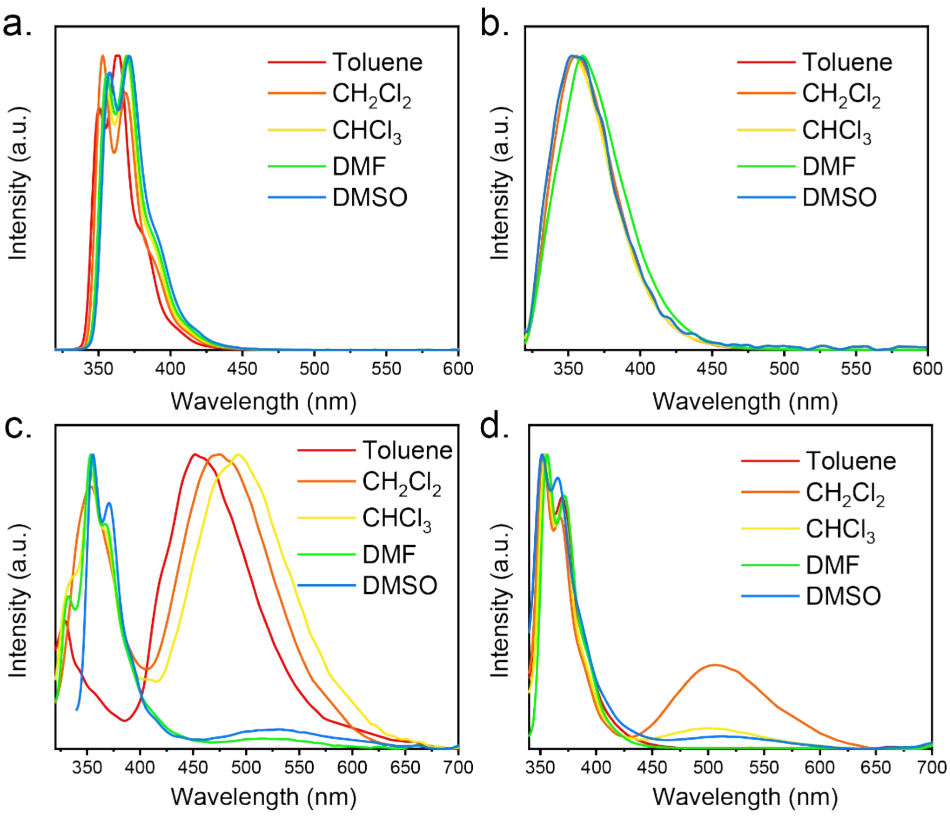

Fig. S3. The prompt PL spectra in various solution (Ex=300 nm) a. **Cz-2C** b. **NP-4C** c. **NP-4C-Cz** d. **NP-6C-Cz**

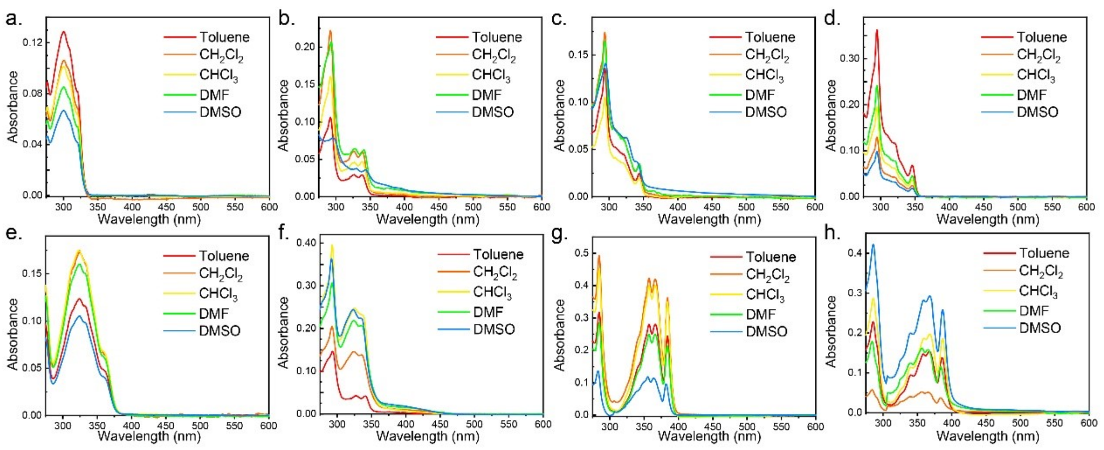

Fig. S4. The UV-visible absorption spectra in various solution 1×10^{-5} M. a. **NP-4C** b.**NP-2C-Cz** c. **NP-4C-Cz** d. **NP-6C-Cz** e. **CN-4C** f. **CN-2C-Cz** g **PY-4C** and h. **PY-2C-Cz**.

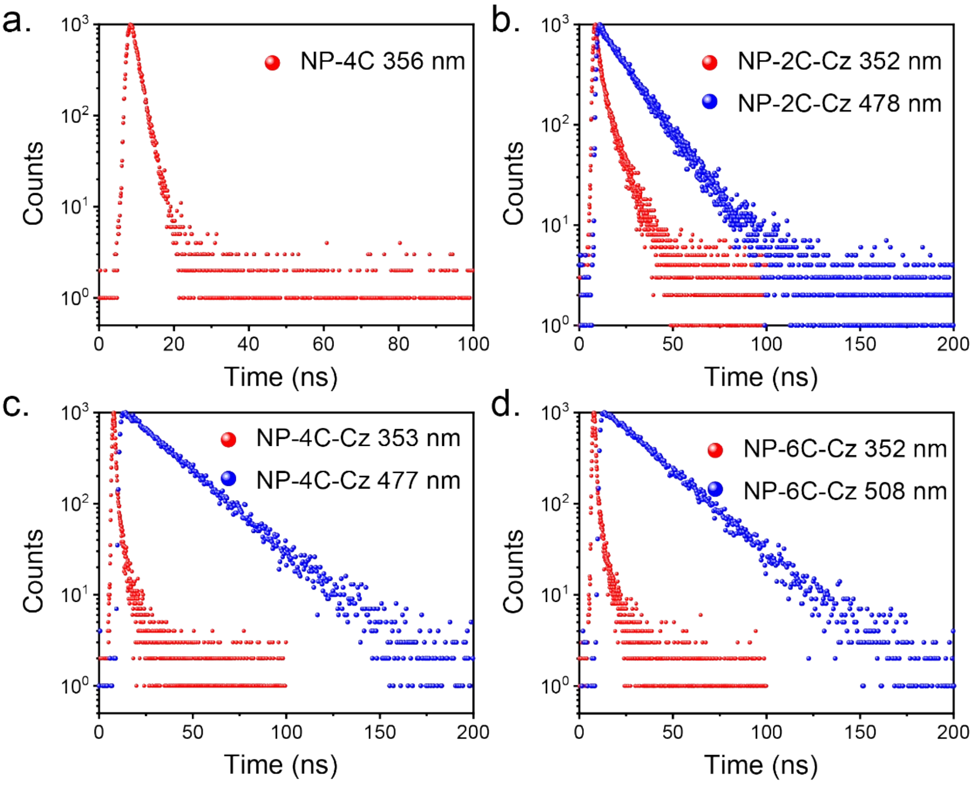

Fig. S5. Fluorescence lifetime decay curve in CH_2Cl_2 1×10^{-5} M a. **NP-4C** b. **NP-2C-Cz** c. **NP-4C-Cz** d. **NP-6C-Cz**.

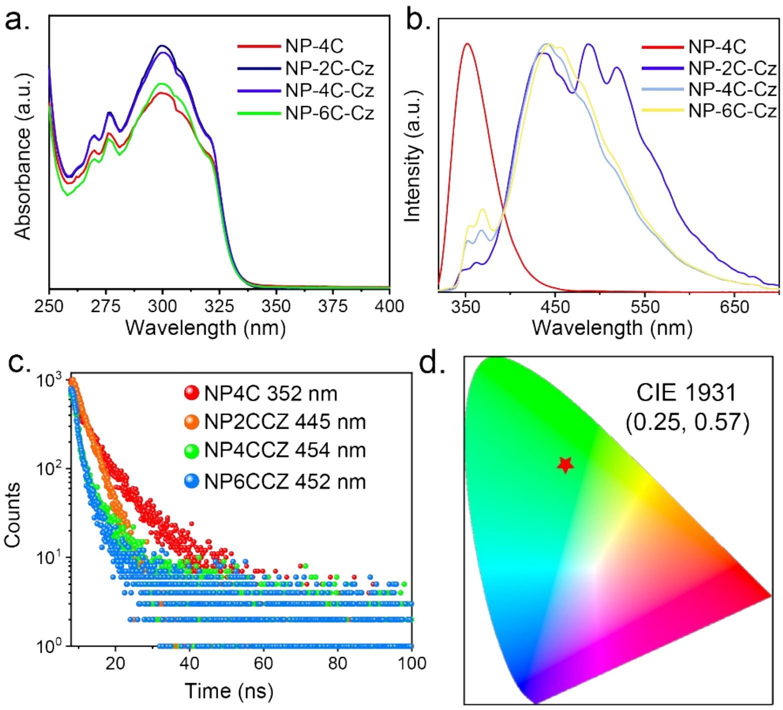

Fig. S6. **NP-4C**, **NP-2C-Cz**, **NP-4C-Cz**, and **NP-6C-Cz** doped PVA films, Wt = 2%. a. The UV-visible absorption spectra. b. The prompt PL spectra (Ex=300 nm) c. Fluorescence lifetime decay curve (Ex = 280 nm). d. CIE chromaticity diagram for **NP-4C**, **NP-2C-Cz**, **NP-4C-Cz**, and **NP-6C-Cz** doped PVA films.

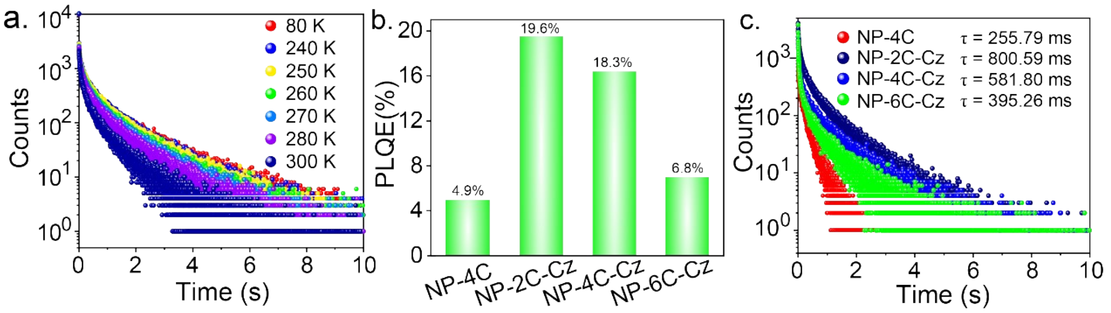

Fig. S7. a. The phosphorescence lifetime decay curve **NP-2C-Cz** doped PVA films at different temperature. **NP-4C**, **NP-2C-Cz**, **NP-4C-Cz**, and **NP-6C-Cz** doped PVA films, Wt = 2%. b. Quantum efficiency comparison of phosphorescence (Ex=300 nm) c. Phos. lifetime decay curve (Ex = 300 nm).

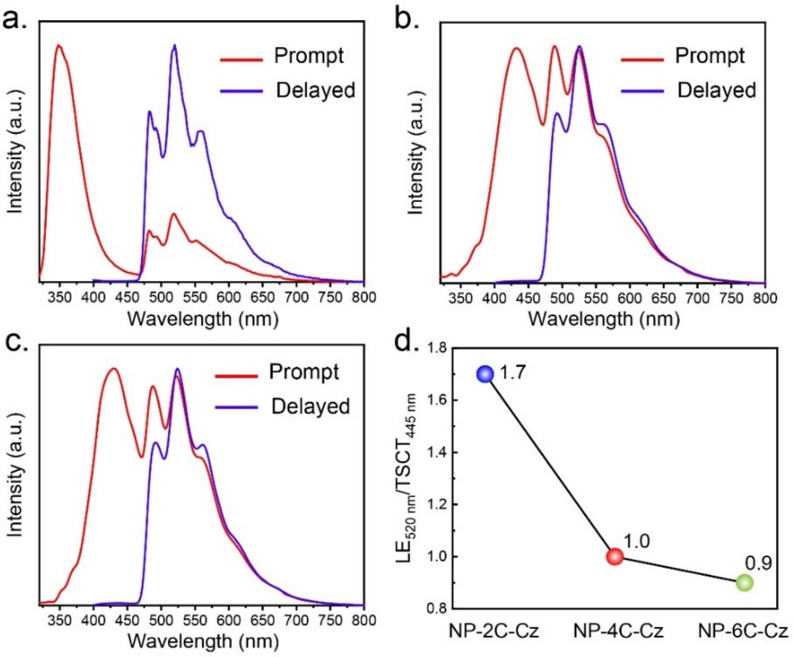

Fig. S8. The prompt and delayed PL spectra at 80 K. a. **NP-4C** b. **NP-4C-Cz** c. **NP-6C-Cz**.

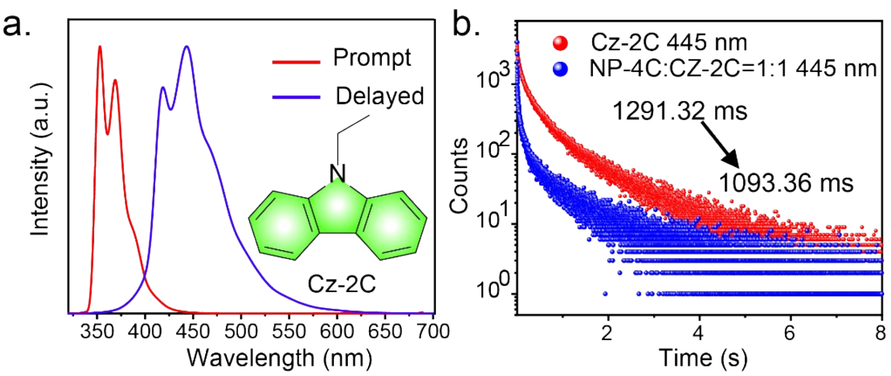

Fig. S9. a. The prompt and delayed spectra of **Cz-2C** doped PVA films. b. Phos. lifetime decay curve of **Cz-2C** and **NP-4C: Cz-2C**=1:1 at 445 nm under 300 nm excitation.

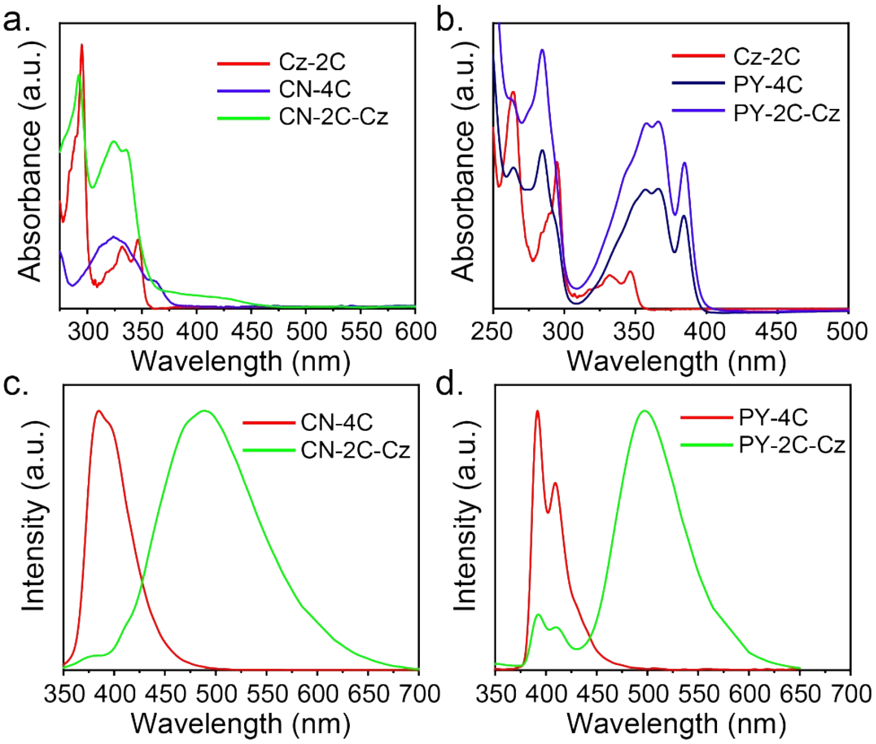

Fig. S10. In CH_2Cl_2 1×10^{-5} M a. The UV-visible absorption spectra of **Cz-2C**, **CN-4C**, and **CN-2C-Cz**. b. The UV-visible absorption spectra of **PY-4C**, and **PY-2C-Cz**. c. The prompt PL spectra of **CN-4C**, and **CN-2C-Cz** (Ex=300 nm). d. The prompt PL spectra of **PY-4C**, and **PY-2C-Cz** (Ex=300 nm).

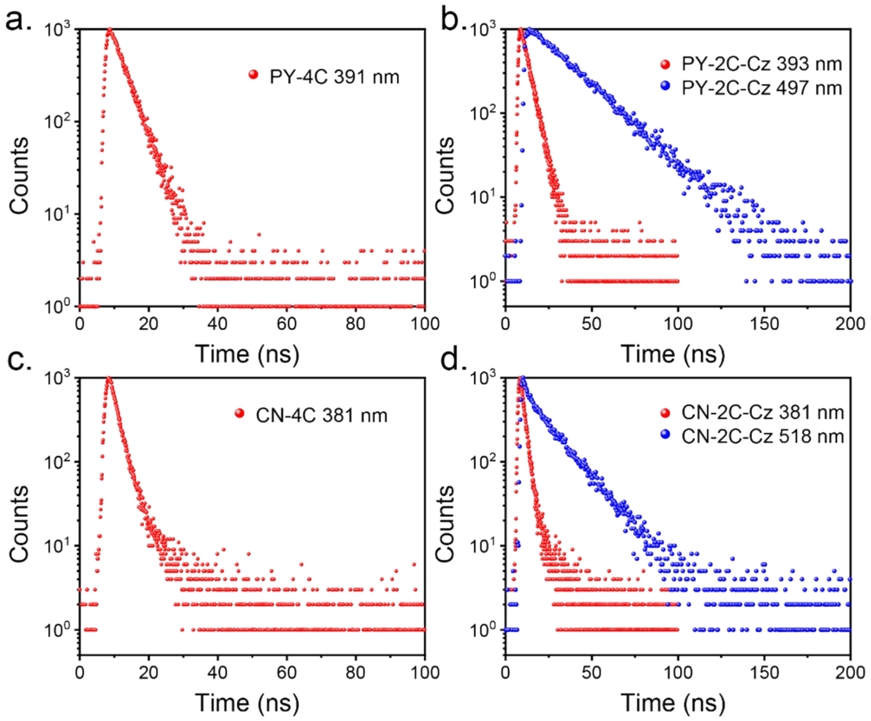

Fig. S11. Fluorescence lifetime decay curve in CH_2Cl_2 1×10^{-5} M (Ex = 280 nm). a. **PY-4C** b. **PY-2C-Cz** c. **CN-4C** d. **CN-2C-Cz**.

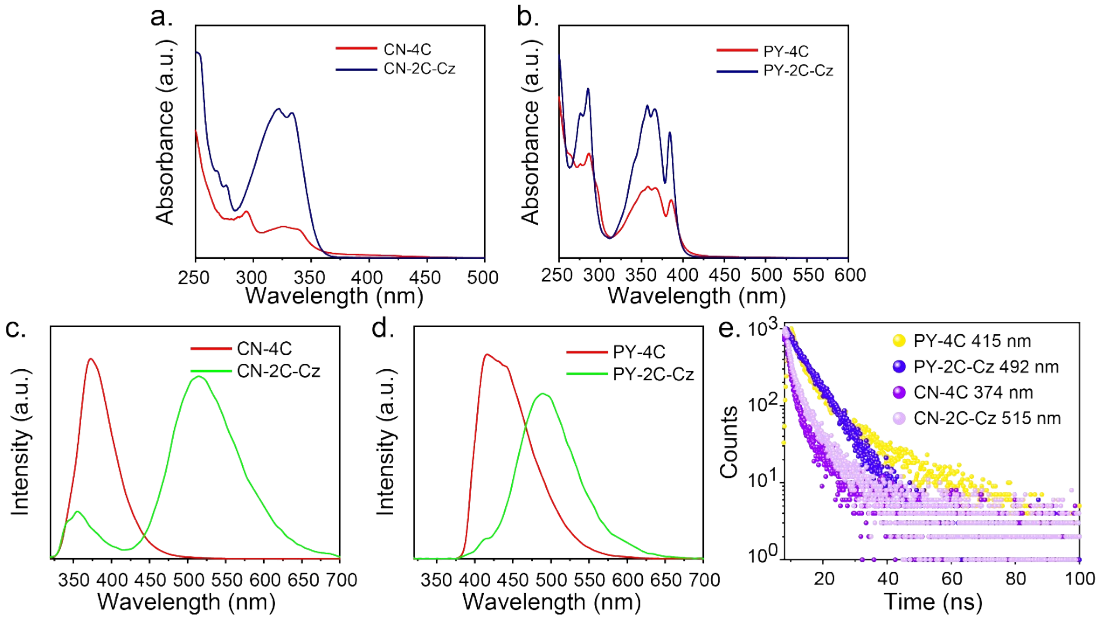

Fig. S12. **CN-4C**, **CN-2C-Cz**, **PY-4C**, and **PY-2C-Cz** doped PVA films, Wt = 2%. a, b the UV-visible absorption spectra. c, d the prompt PL spectra (Ex=300 nm) e. Fluorescence lifetime decay curve (Ex = 280 nm).

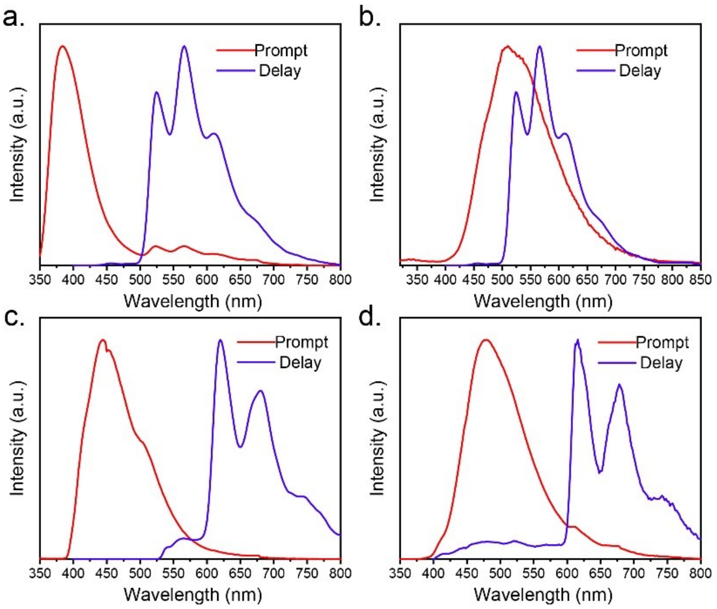

Fig. S13. The prompt and delayed PL spectra at 80 K. a. **CN-4C** b. **CN-2C-Cz** c. **PY-4C** d. **PY-2C-Cz**

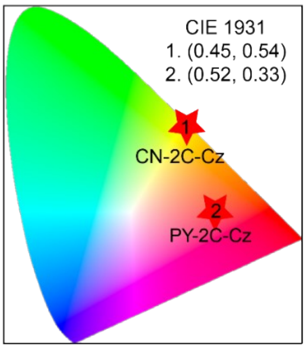

Fig. S14. CIE chromaticity diagram for **CN-4C**, **CN-2C-Cz**, **PY-4C**, and **PY-2C-Cz** doped PVA films.

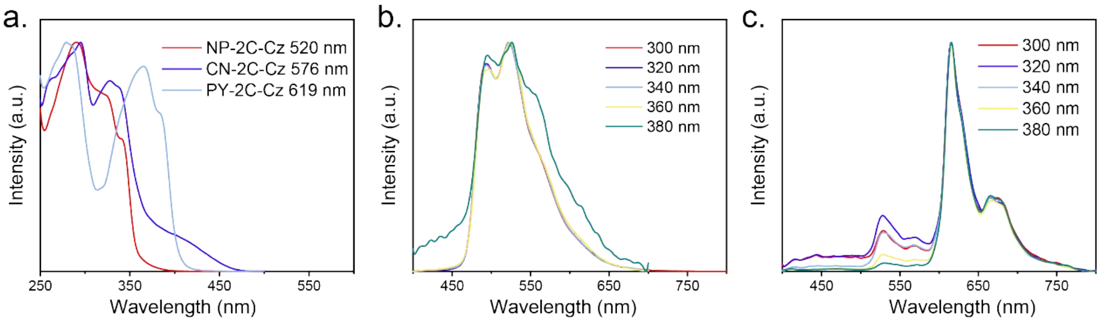

Fig. S15. a. The excitation spectra of **NP-2C-Cz** and **PY-2C-Cz** doped PVA films at wavelengths of 520 nm and 619 nm, respectively. b. The delayed PL spectra of **NP-2C-Cz** doped PVA films at various excitation. c. The delayed PL spectra of **PY-2C-Cz** doped PVA films at various excitation.

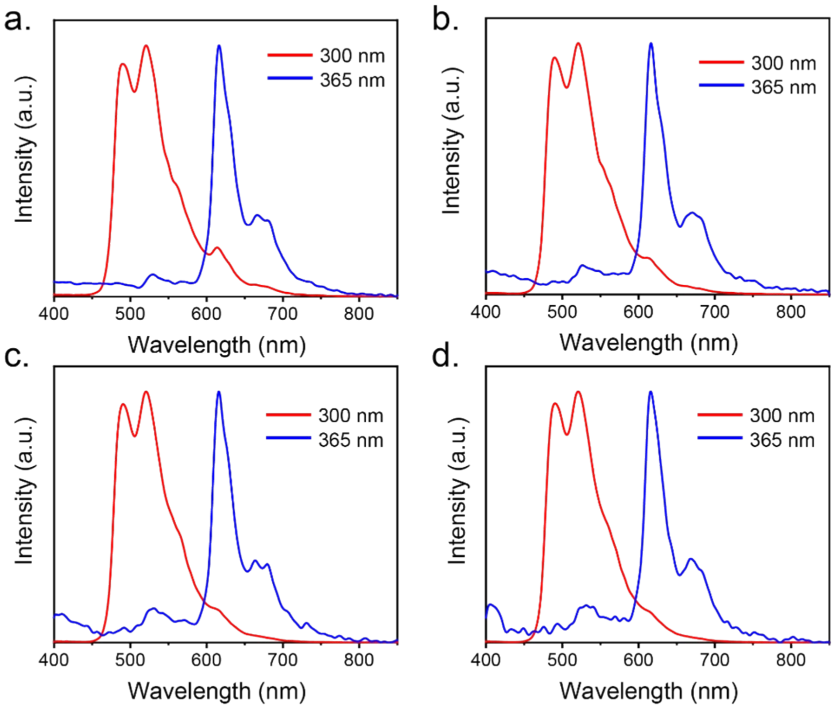

Fig. S16. The delayed spectra of PVA films with different doping ratios of **NP-2C-Cz**: **PY-2C-Cz**. a. 5:1 b. 10:1 c. 15 :1 d. 20:1.

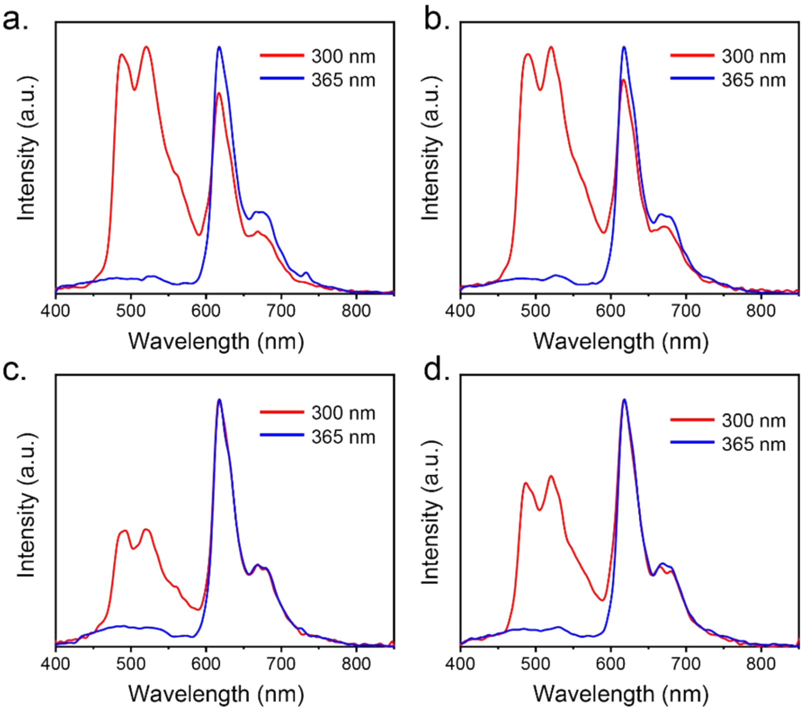

Fig. S17. The delayed spectra of PVA films with different doping ratios of **PY-2C-Cz**: **NP-2C-Cz**. a. 5:1 b. 10:1 c. 15 :1 d. 20:1.

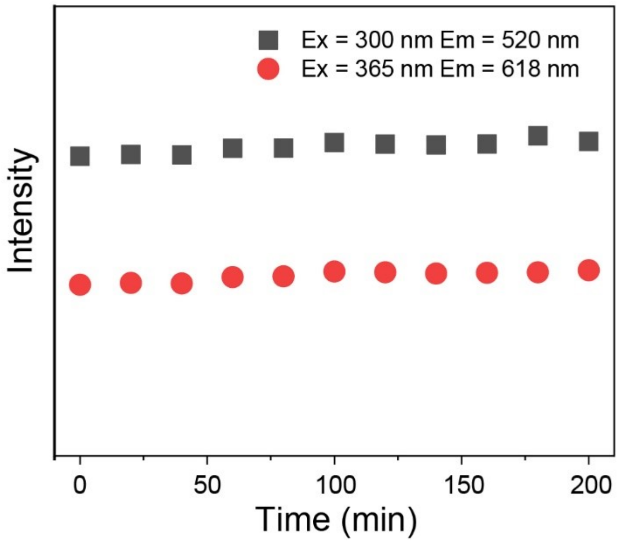

Fig. S18. The photostability of PVA films doped with a 5:2 ratio mixture of **NP-2C-Cz** and **PY-2C-Cz**.

4. Characterization data

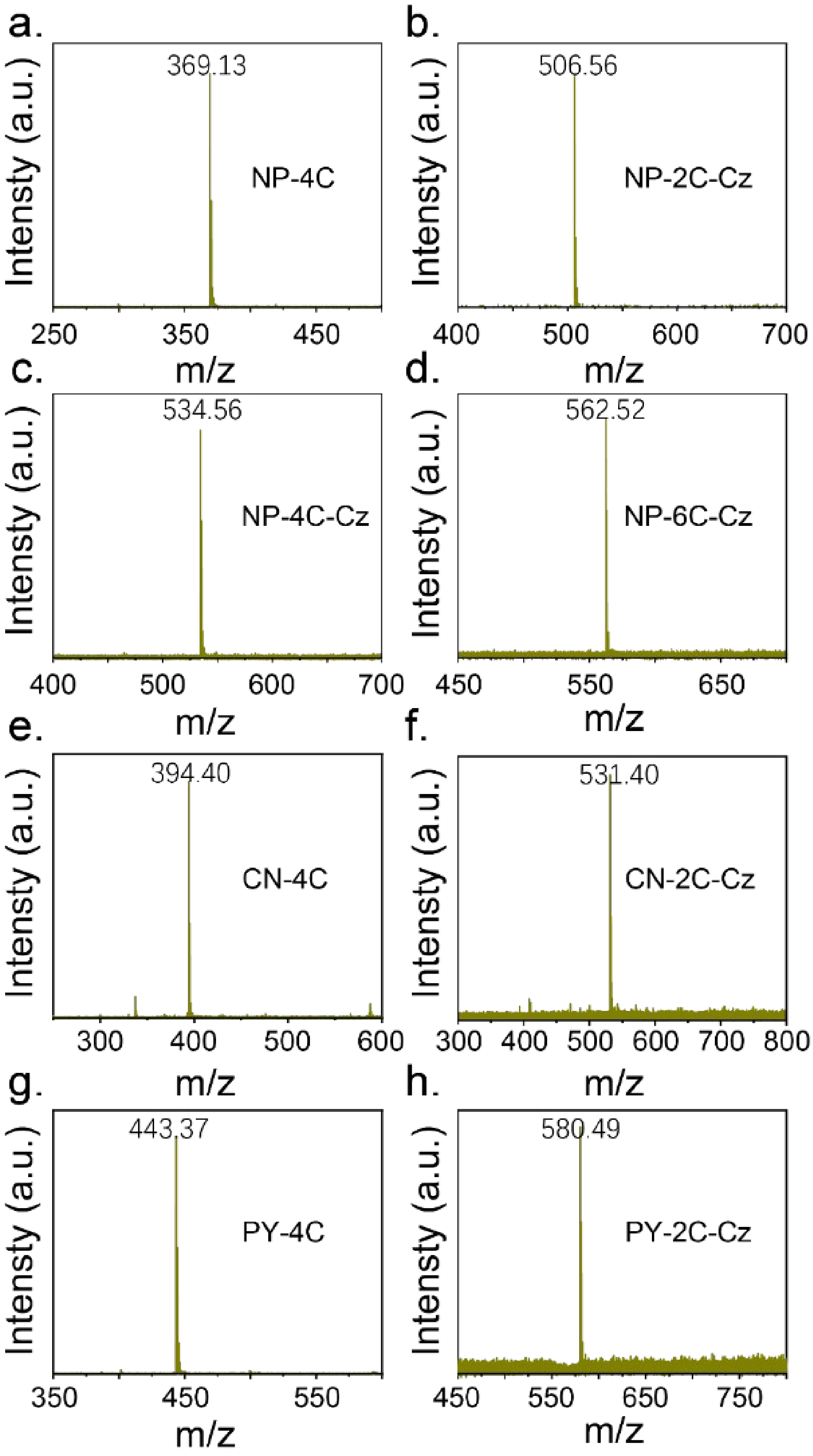

Fig. S19. MALDI-TOF spectra. a. **NP-4C** b. **NP-2C-Cz** c. **NP-4C-Cz** d. **NP-6C-Cz** e. **CN-4C** f. **CN-2C-Cz** g. **PY-4C** h. **PY-2C-Cz**

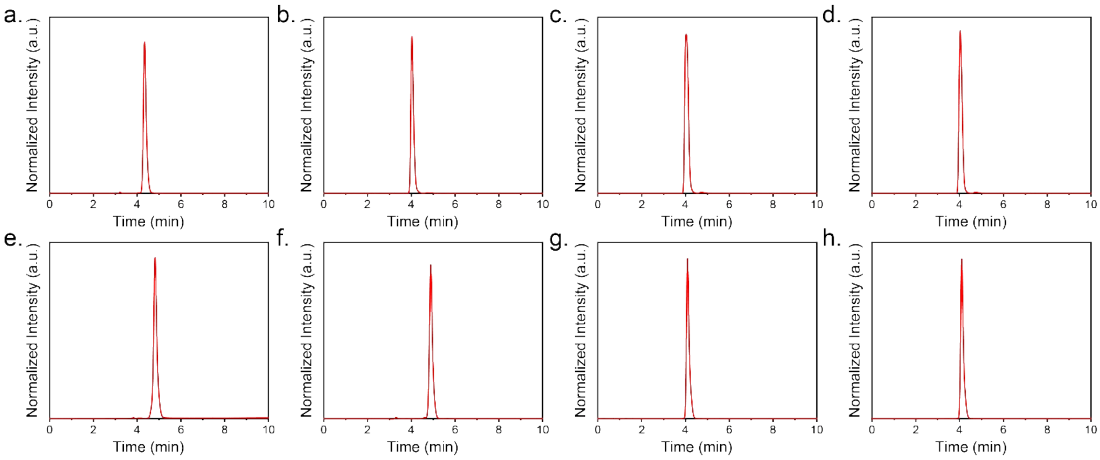

Fig. S20. HPLC spectra. a. **NP-4C** b. **NP-2C-Cz** c. **NP-4C-Cz** d. **NP-6C-Cz** e. **CN-4C** f. **CN-2C-Cz** g. **PY-4C** h. **PY-2C-Cz**

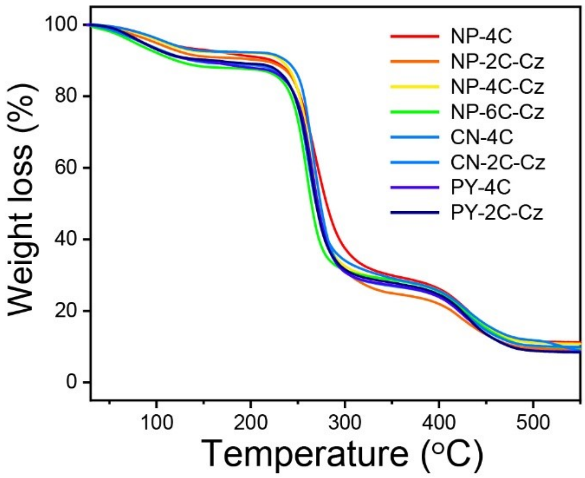

Fig. S21. TGA curves of **NP-4C**, **NP-2C-Cz**, **NP-4C-Cz**, **NP-6C-Cz**, **CN-4C**, **CN-2C-Cz**, **PY-4C** and **PY-2C-Cz** doped PVA films.

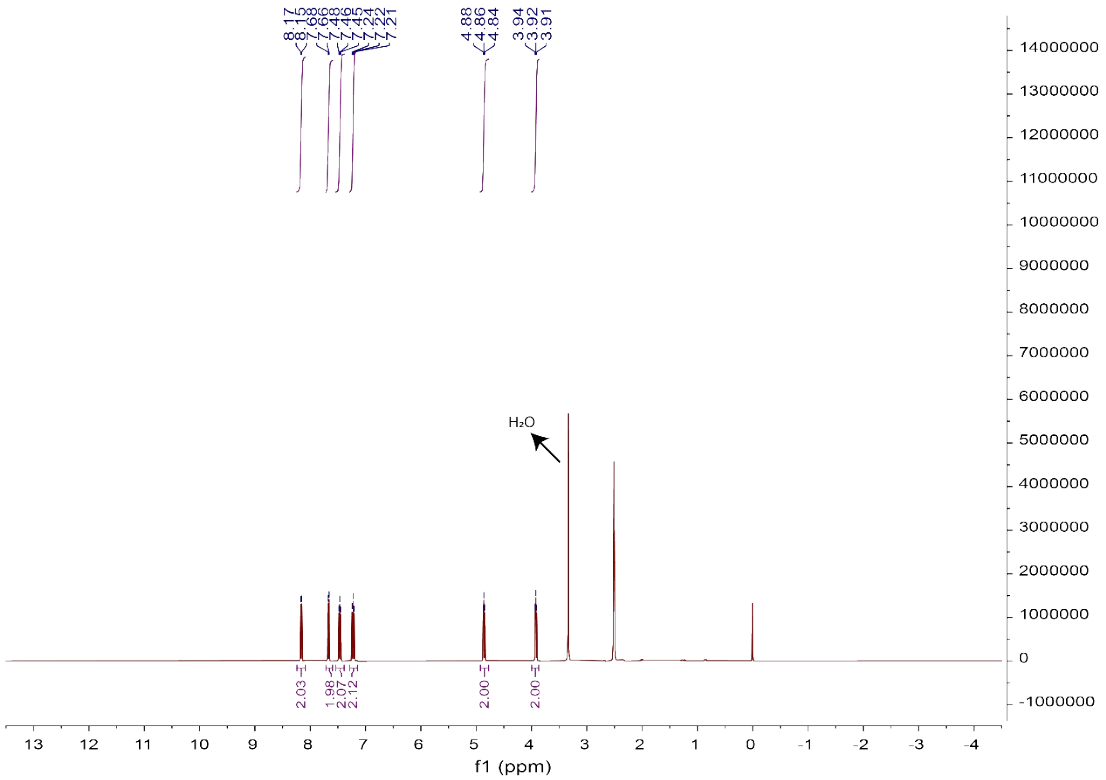

Fig. S22. ^{1}H NMR spectra of **9-(2-bromoethyl)-9H-carbazole** in DMSO-d_6

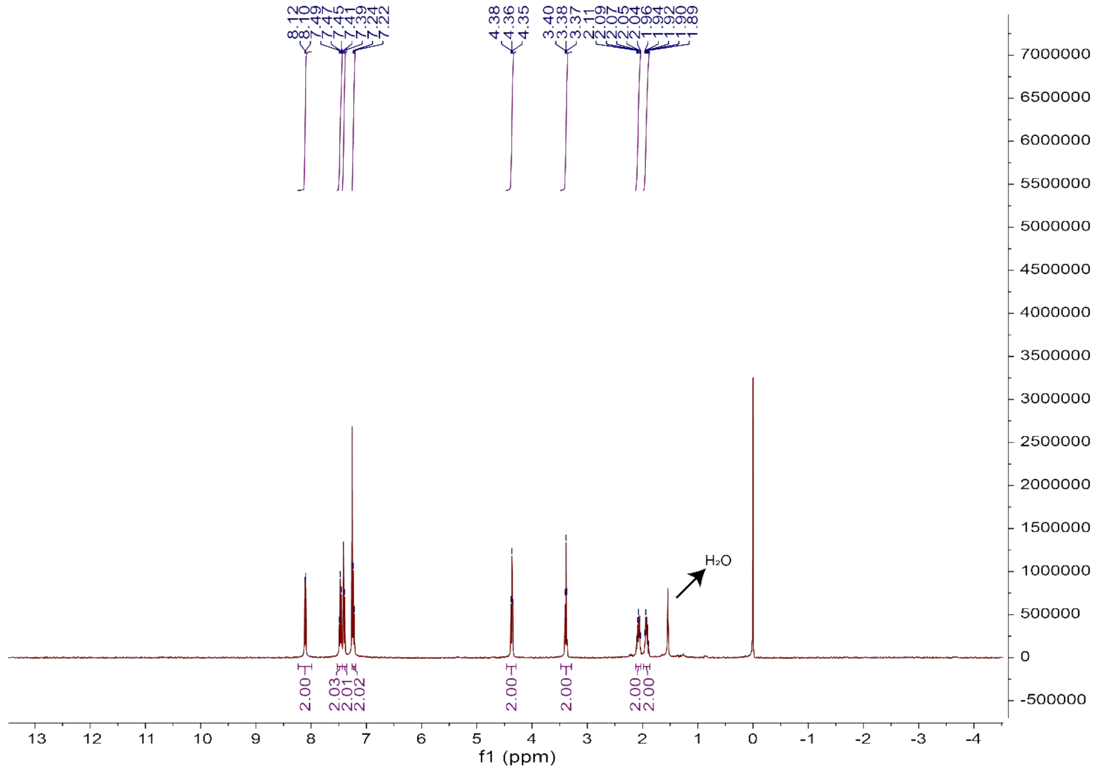

Fig. S23. ^{1}H NMR spectra of **9-(4-bromobutyl)-9H-carbazole** in DMSO-d_6

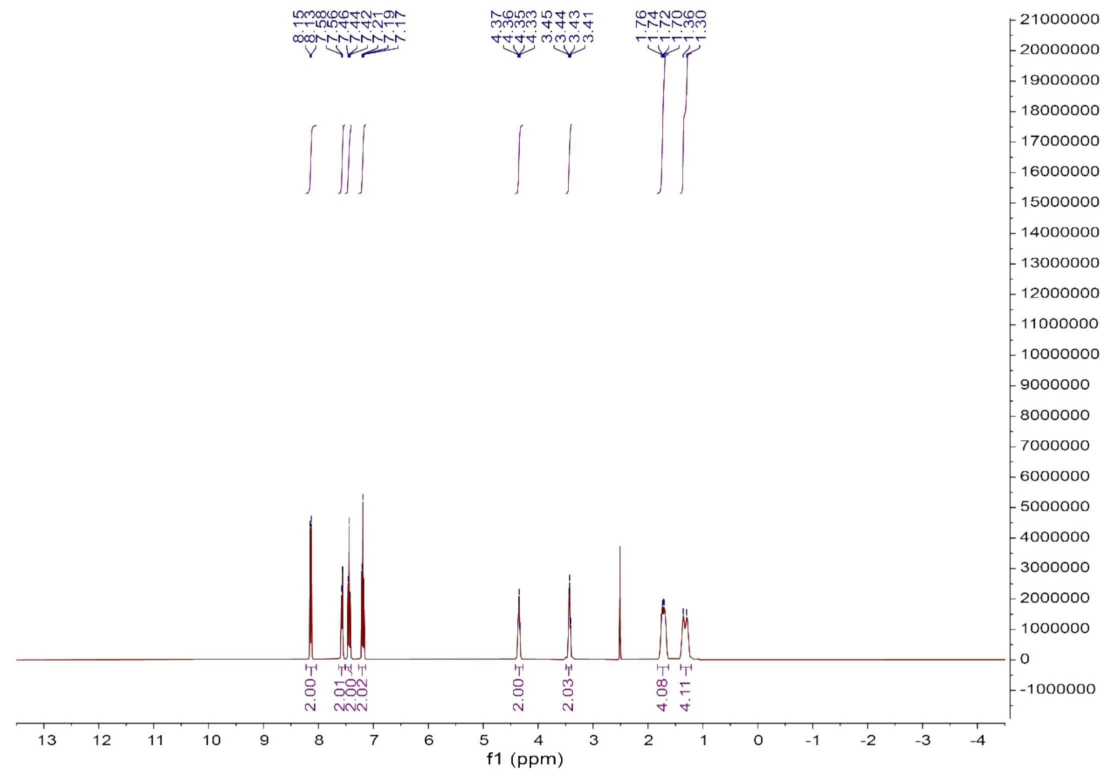

Fig. S24. ^{1}H NMR spectra of **9-(6-bromohexyl)-9H-carbazole** in DMSO-d_6

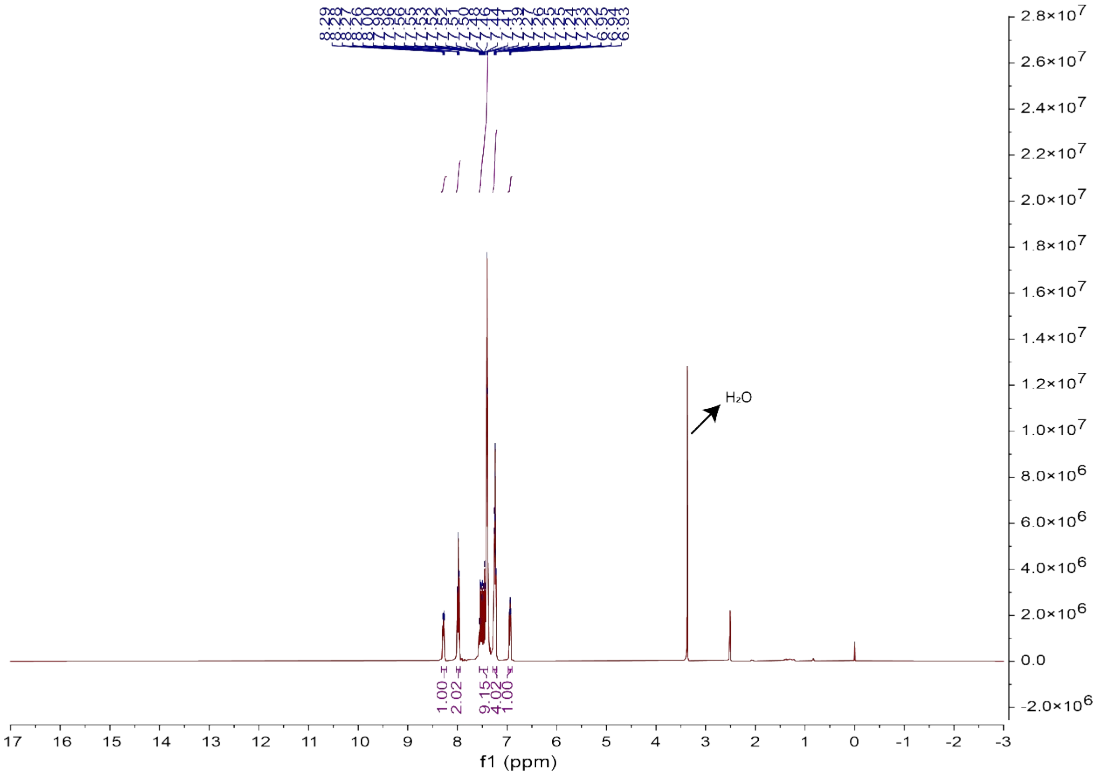

Fig. S25. ^{1}H NMR spectra of **Naphthalen-1-yldiphenylphosphane** in DMSO-d_6

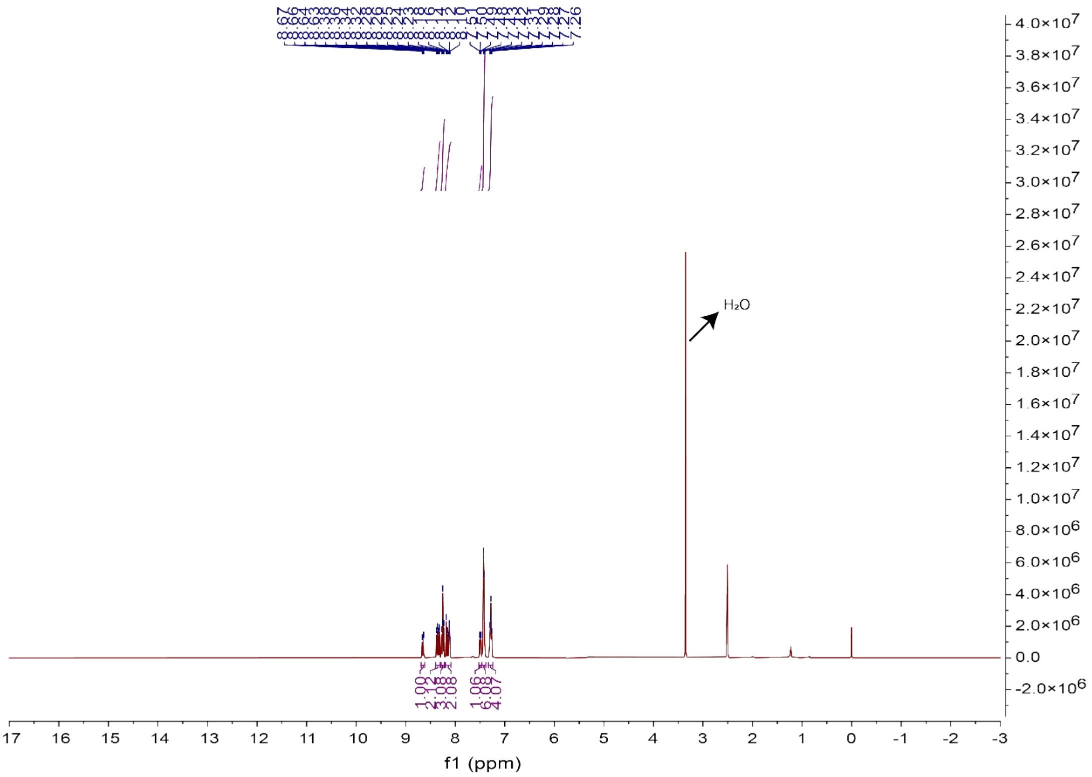

Fig. S26. ^{1}H NMR spectra of **Diphenyl(pyren-1-yl)phosphane** in DMSO-d_6

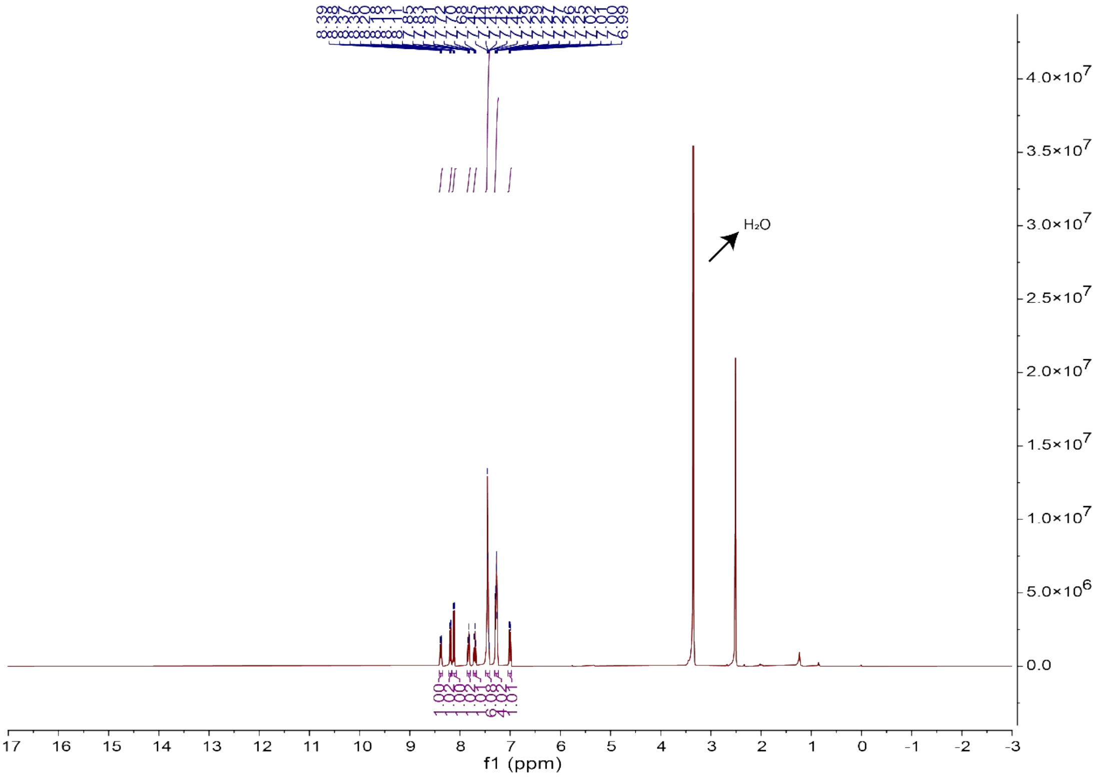

Fig. S27. ^{1}H NMR spectra of **4-(diphenylphosphaneyl)-1-naphthonitrile** in DMSO-d_6

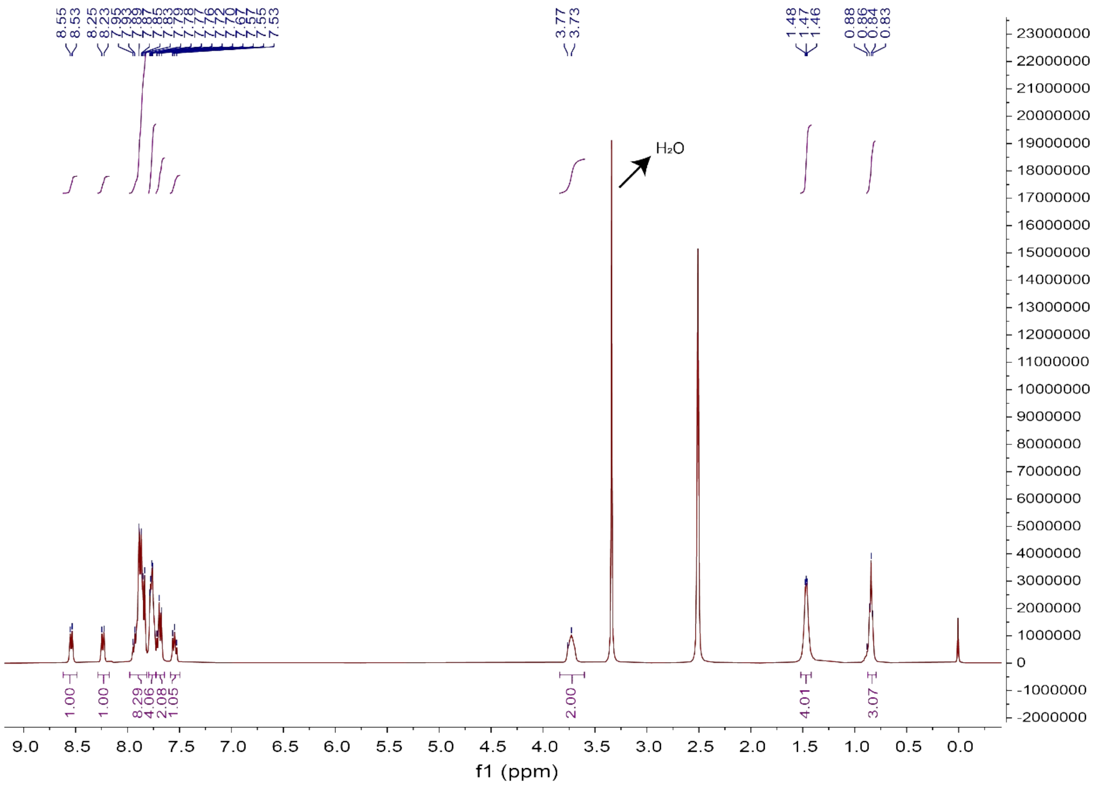

Fig. S28. ^{1}H NMR spectra of **NP-4C** in DMSO-d_6

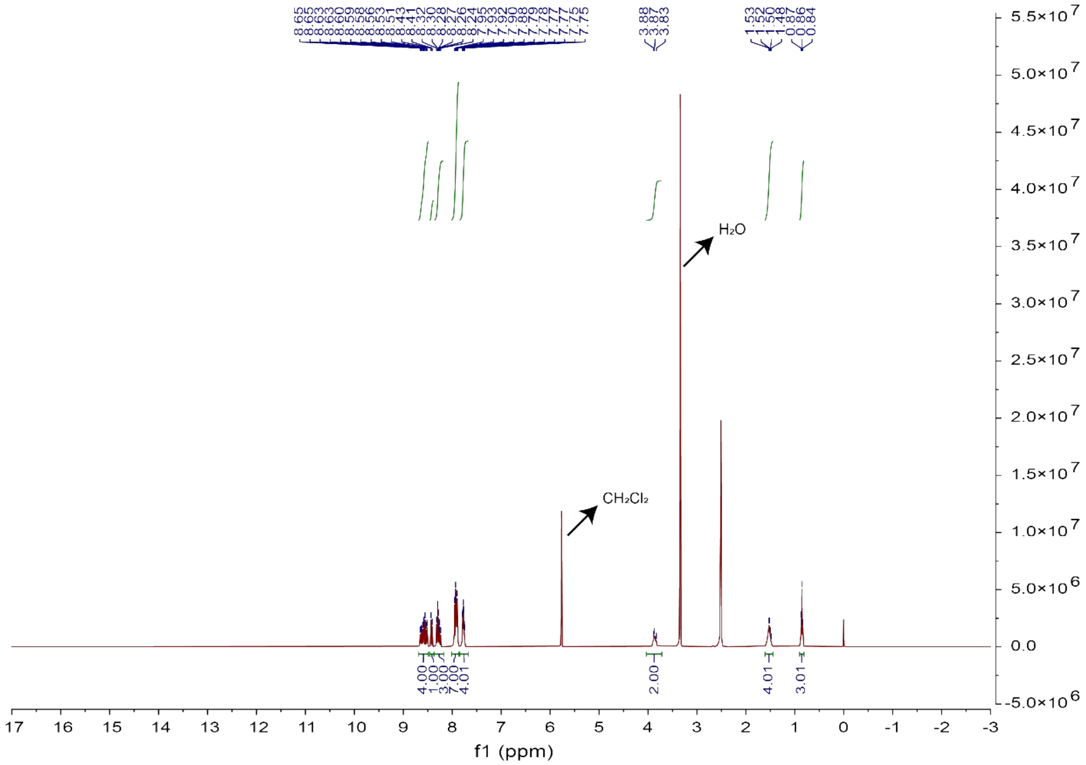

Fig. S29. ^{1}H NMR spectra of **PY-4C** in DMSO-d_6

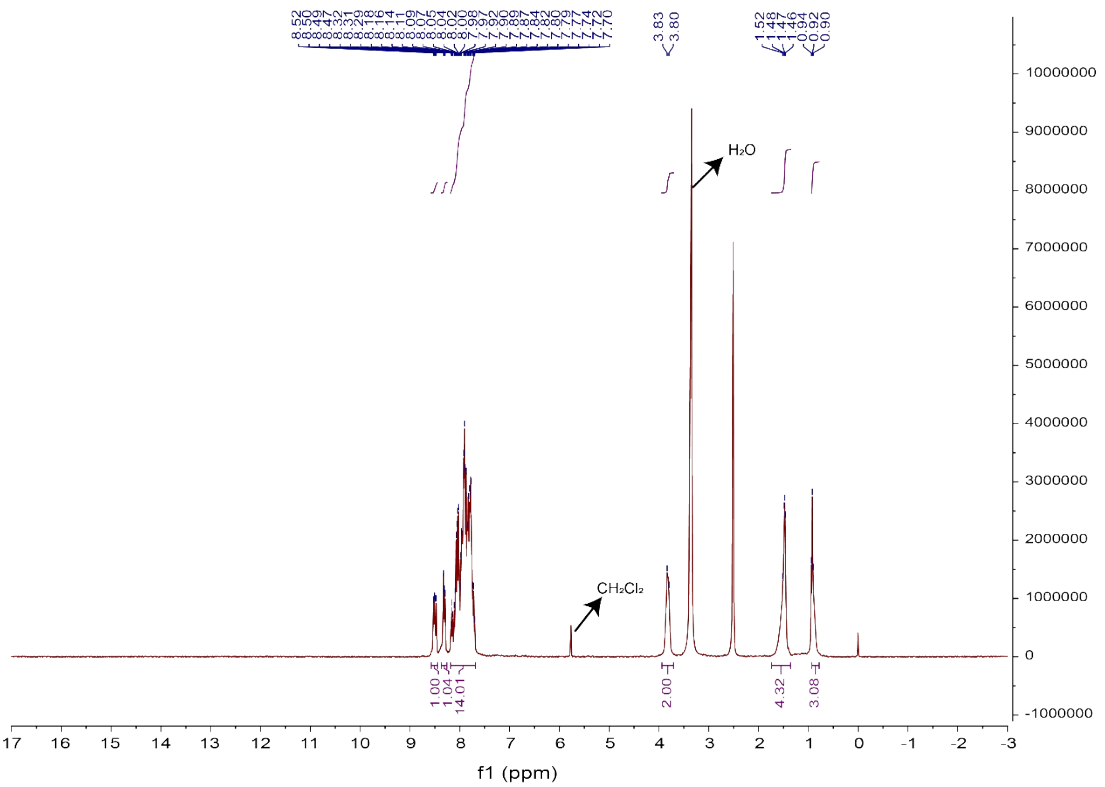

Fig. S30. ^{1}H NMR spectra of **CN-4C** in DMSO-d_6

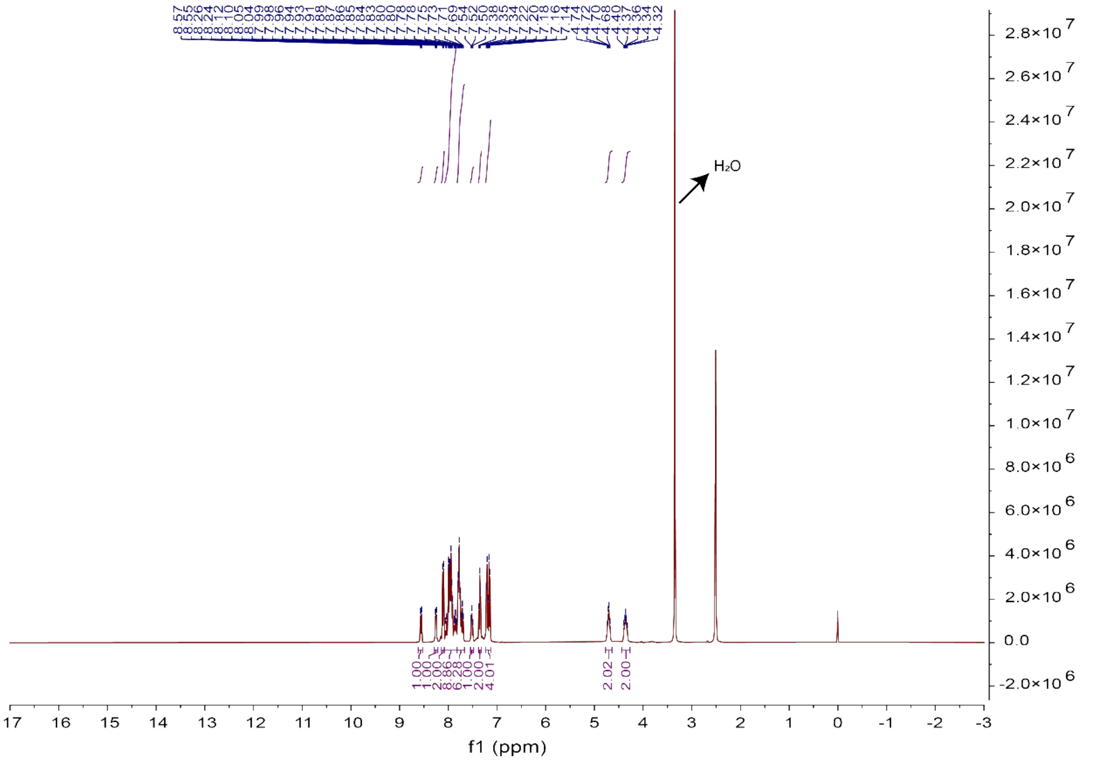

Fig. S31. ^{1}H NMR spectra of **NP-2C-Cz** in DMSO-d_6

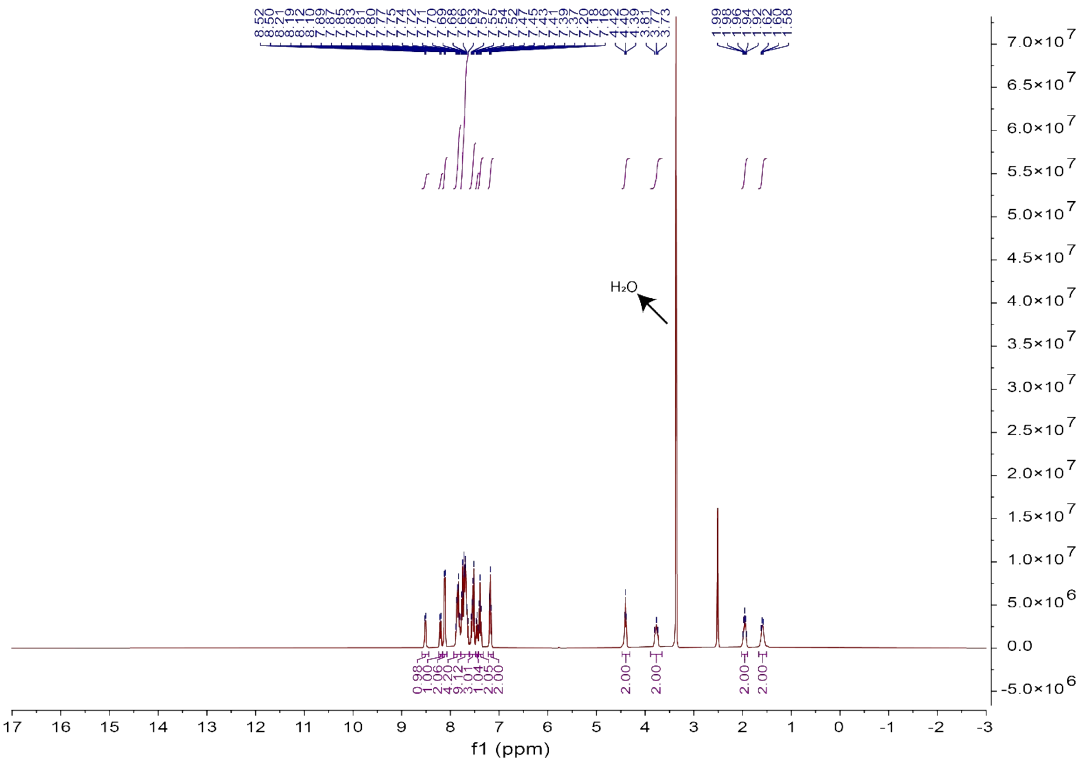

Fig. S32. ^{1}H NMR spectra of **NP-4C-Cz** in DMSO-d_6

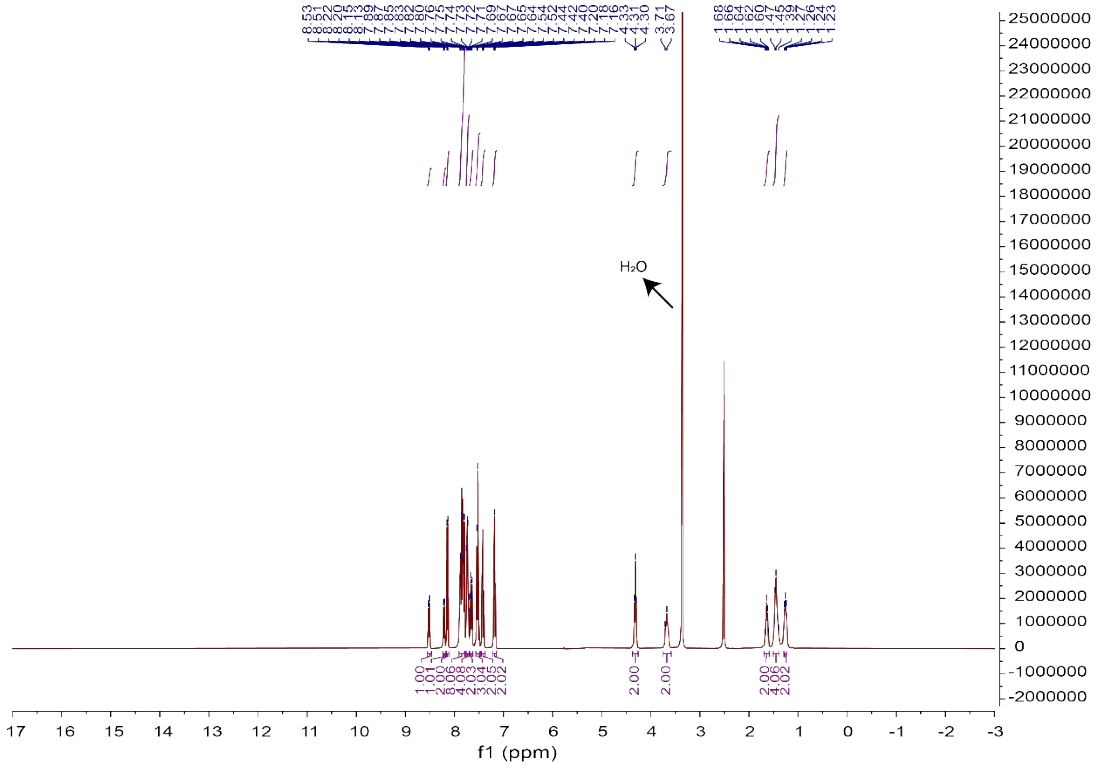

Fig. S33. ^{1}H NMR spectra of **NP-6C-Cz** in DMSO-d_6

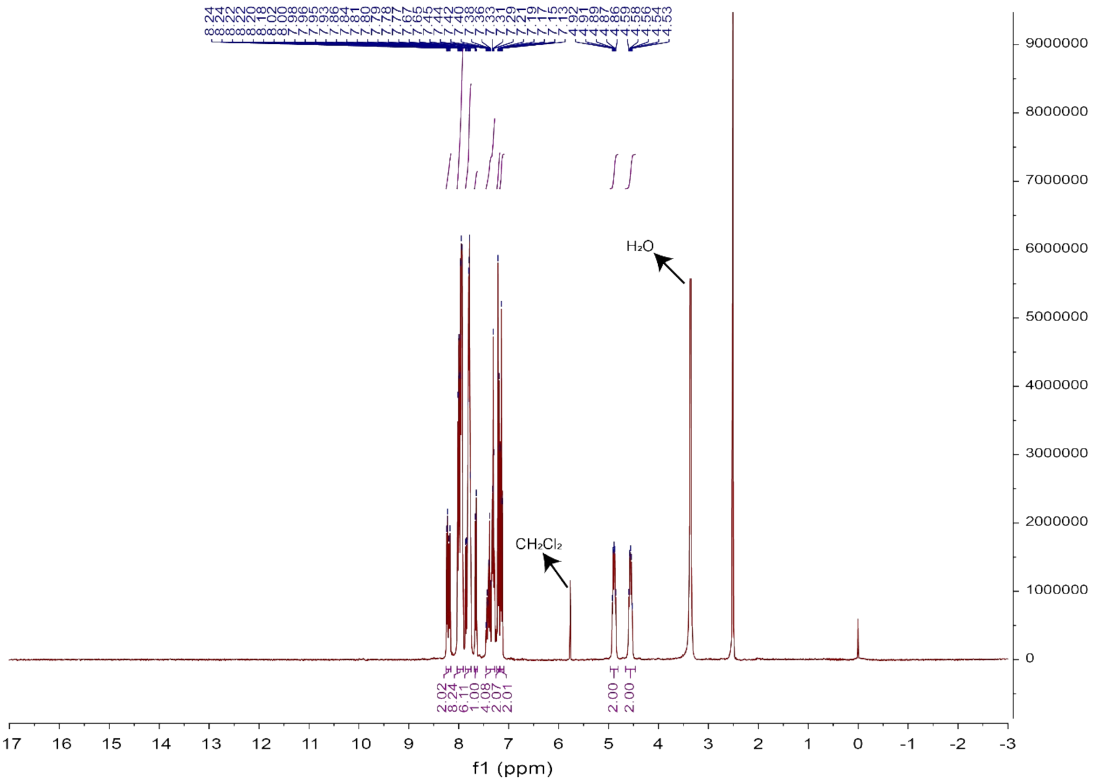

Fig. S34. ^{1}H NMR spectra of **CN-2C-Cz** in DMSO-d_6

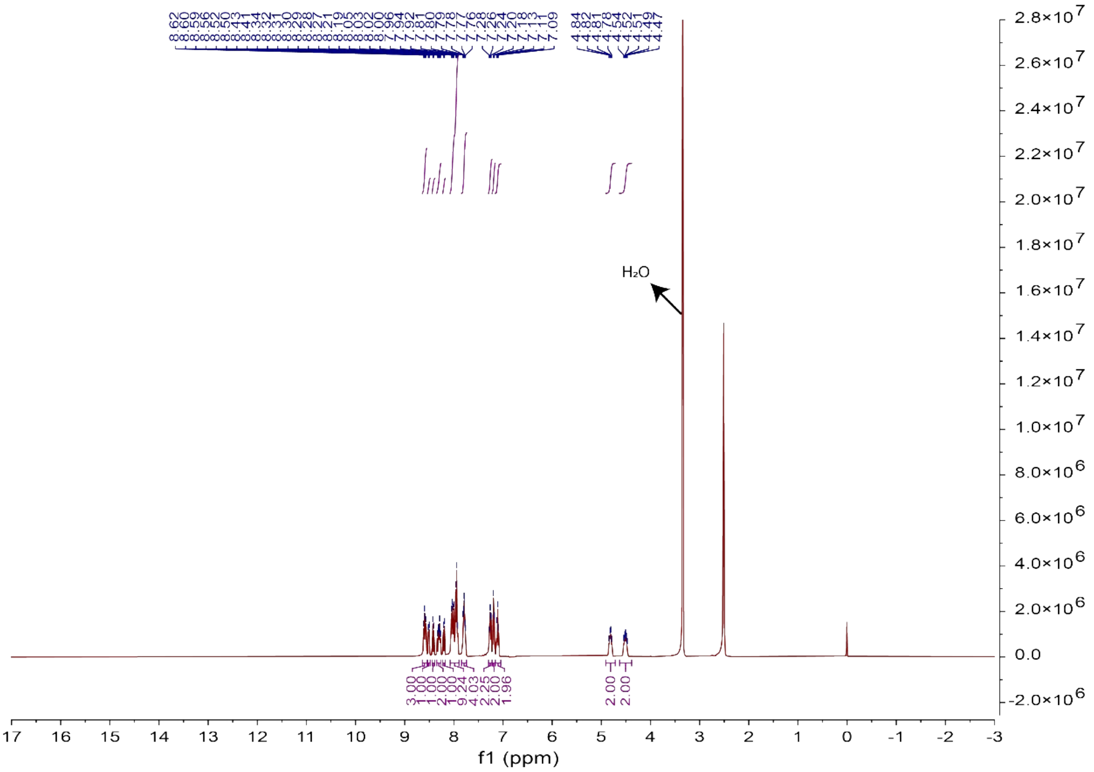

Fig. S35. ^{1}H NMR spectra of **PY-2C-Cz** in DMSO-d_6

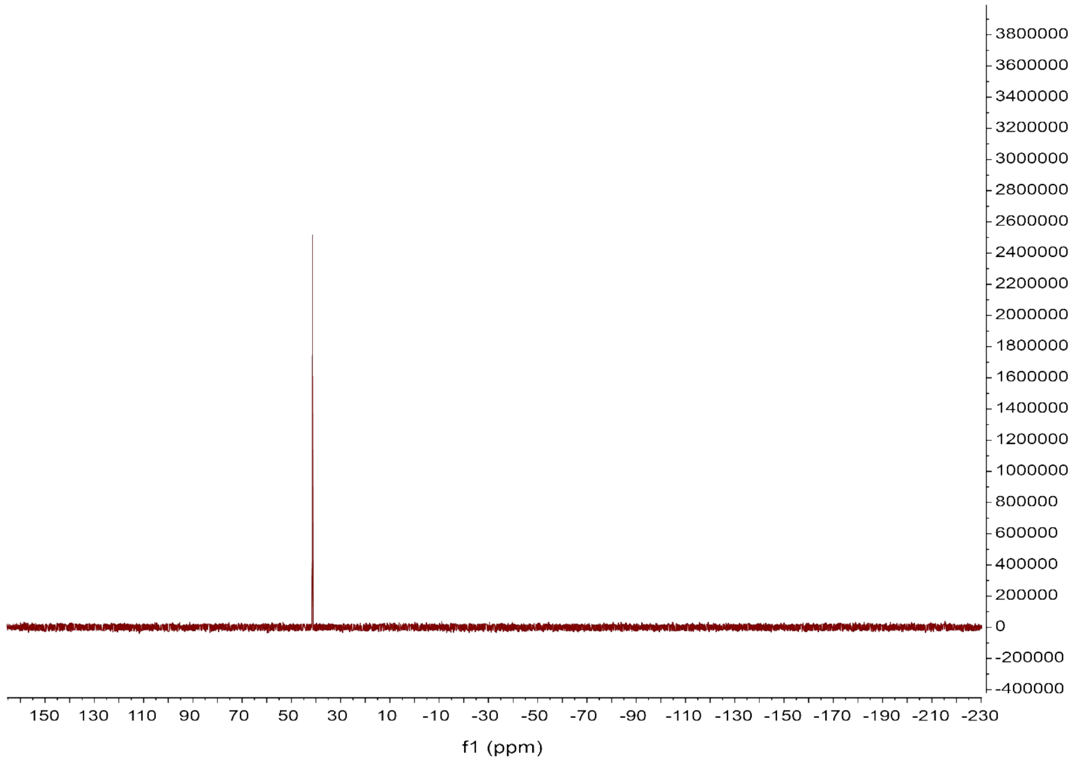

Fig. S36. ^{31}P NMR spectra of **NP-4C** in DMSO-d_6

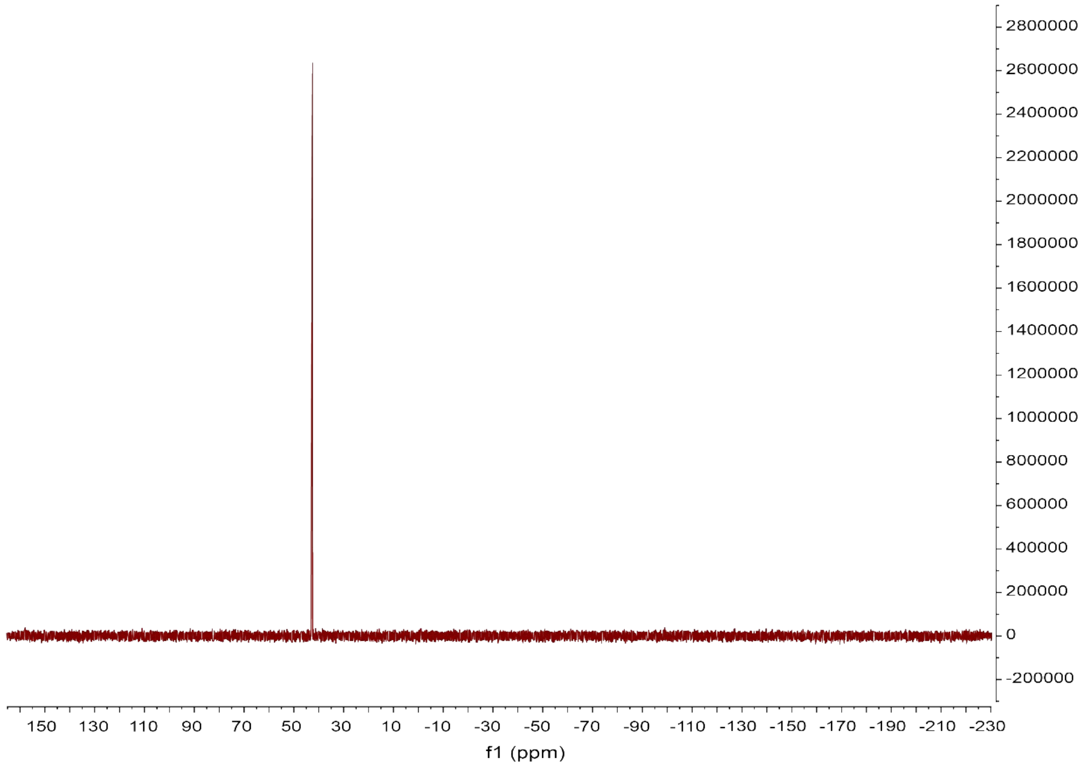

Fig. S37. ^{31}P NMR spectra of **CN-4C** in DMSO-d_6

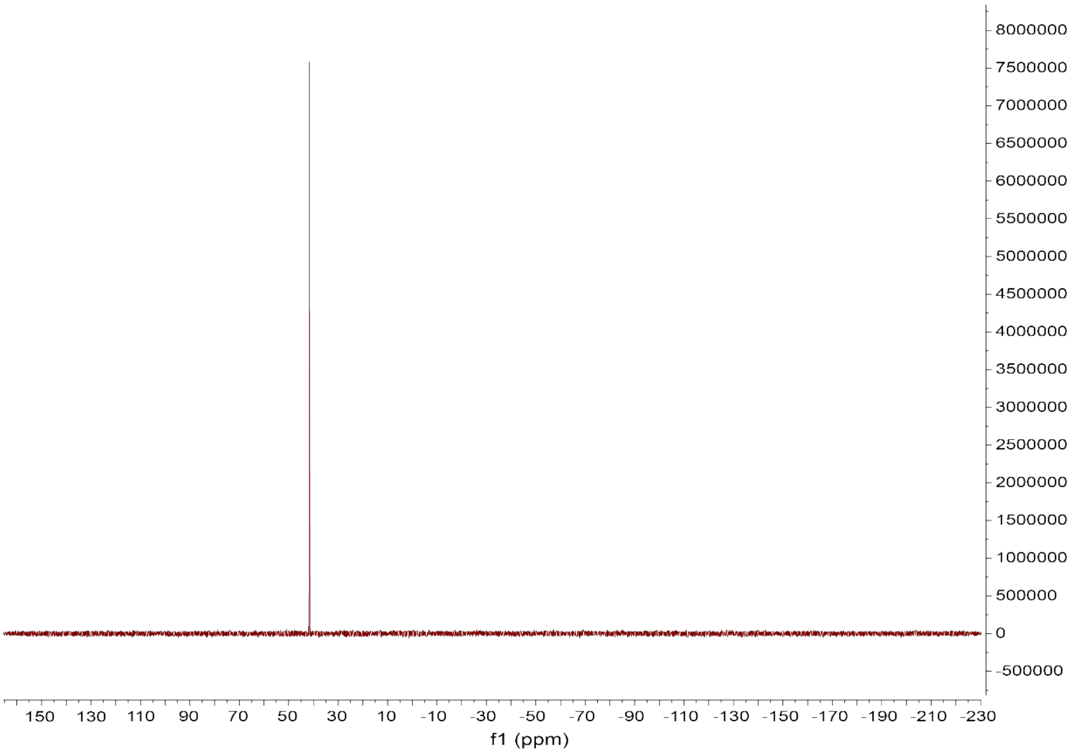

Fig. S38. ^{31}P NMR spectra of **PY-4C** in DMSO-d_6

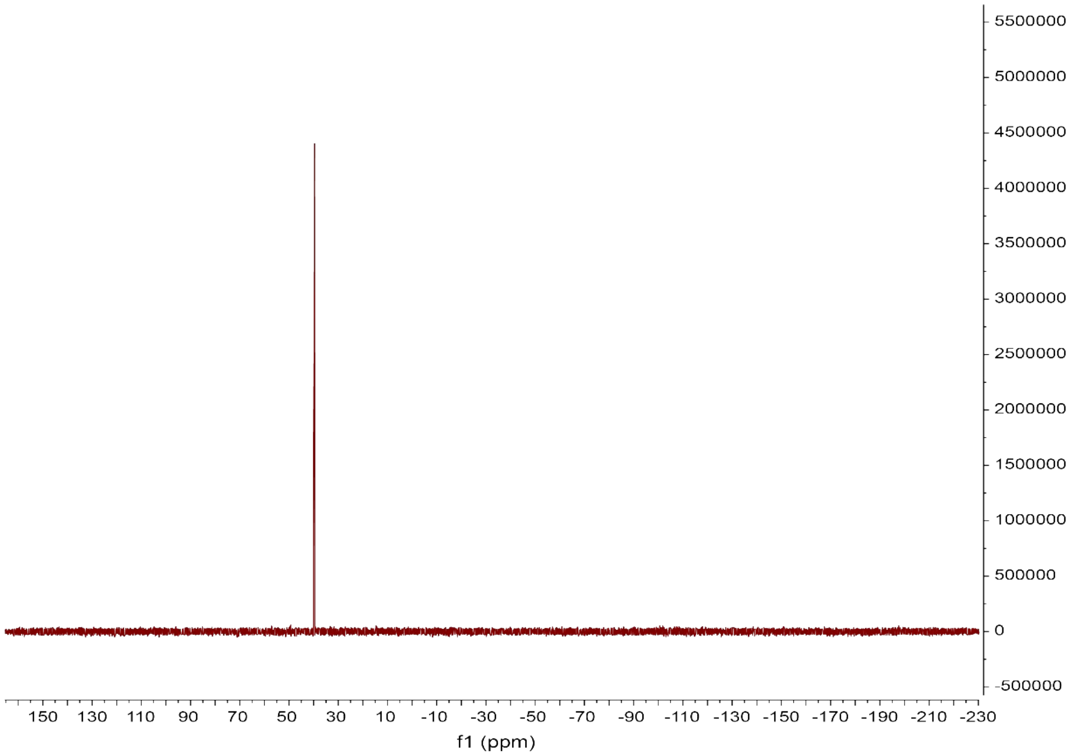

Fig. S39. ^{31}P NMR spectra of **NP-2C-Cz** in DMSO-d_6

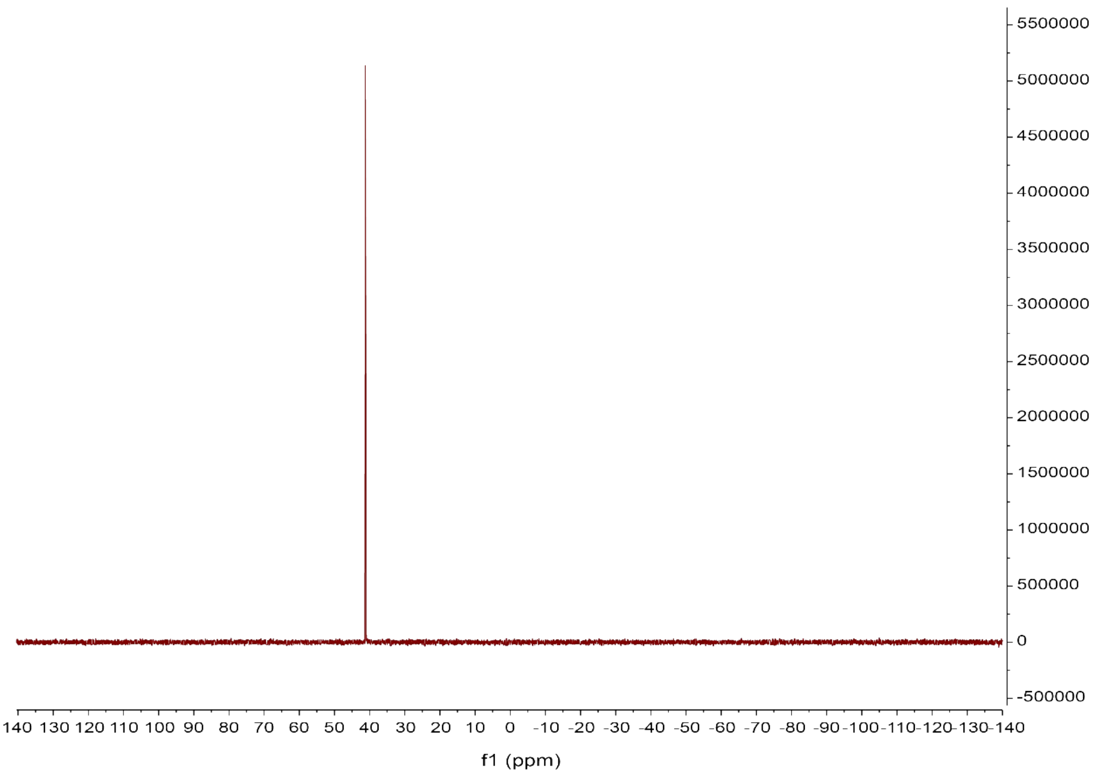

Fig. S40. ^{31}P NMR spectra of **NP-4C-Cz** in DMSO-d_6

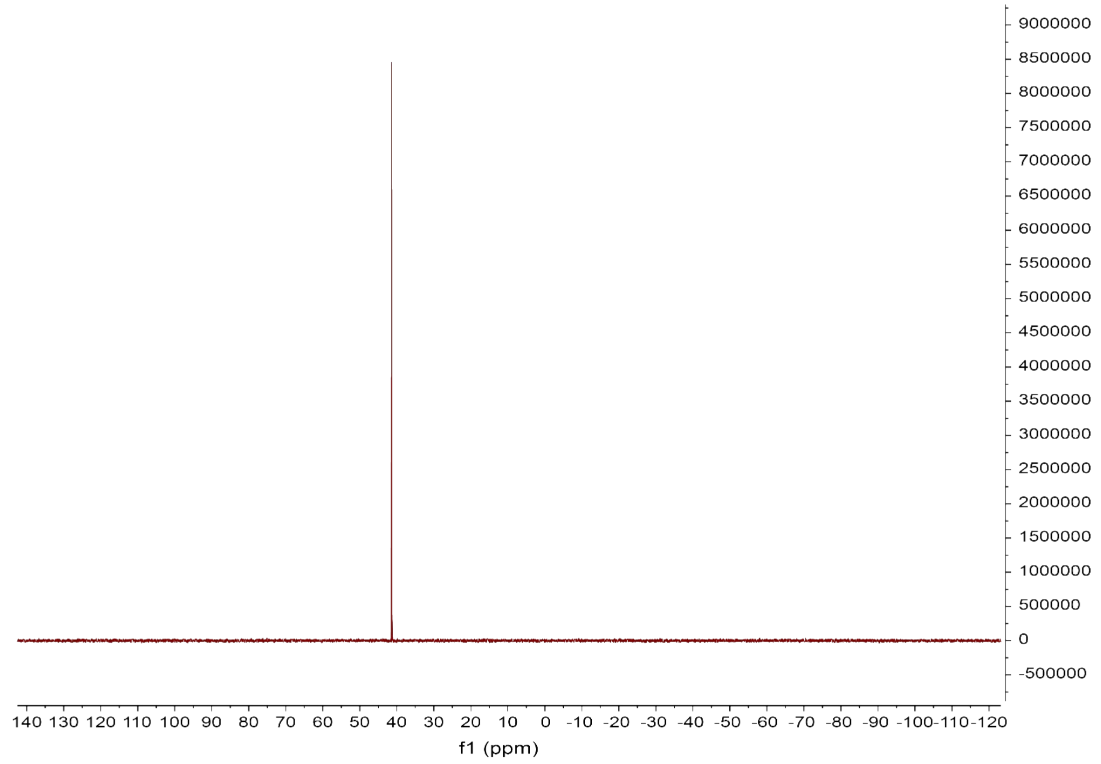

Fig. S41. ^{31}P NMR spectra of **NP-6C-Cz** in DMSO-d_6

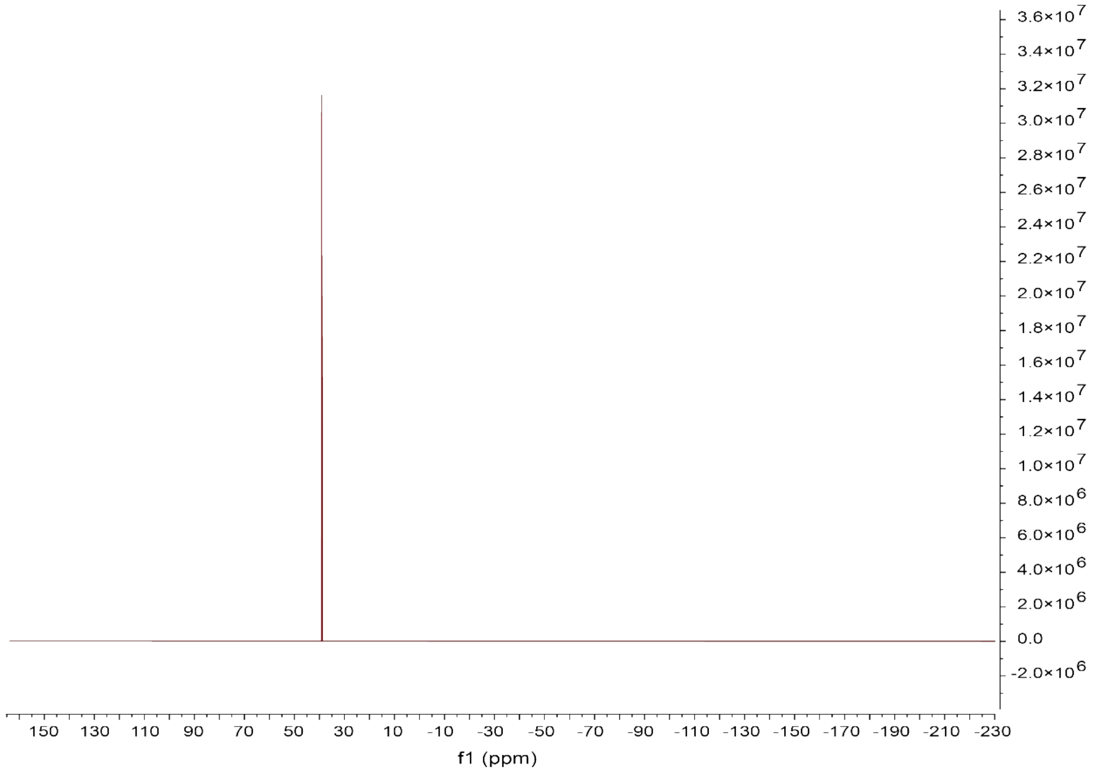

Fig. S42. ^{31}P NMR spectra of **CN-2C-Cz** in DMSO-d_6

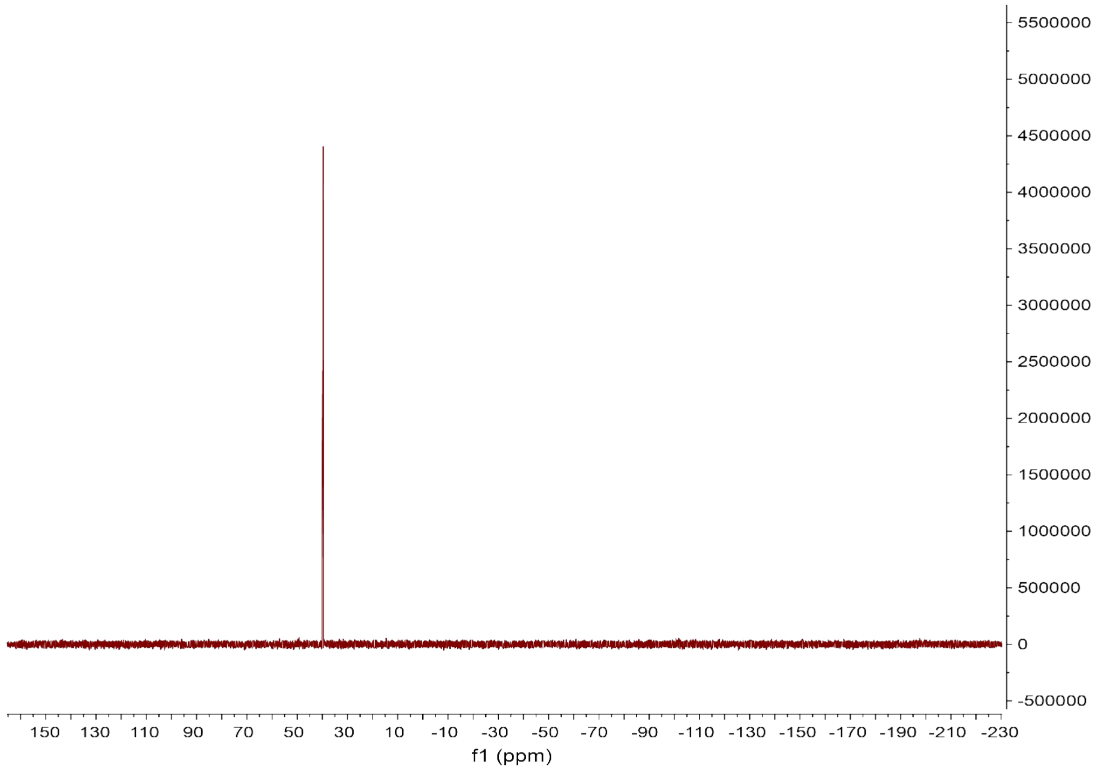

Fig. S43. ^{31}P NMR spectra of **PY-2C-Cz** in DMSO-d_6

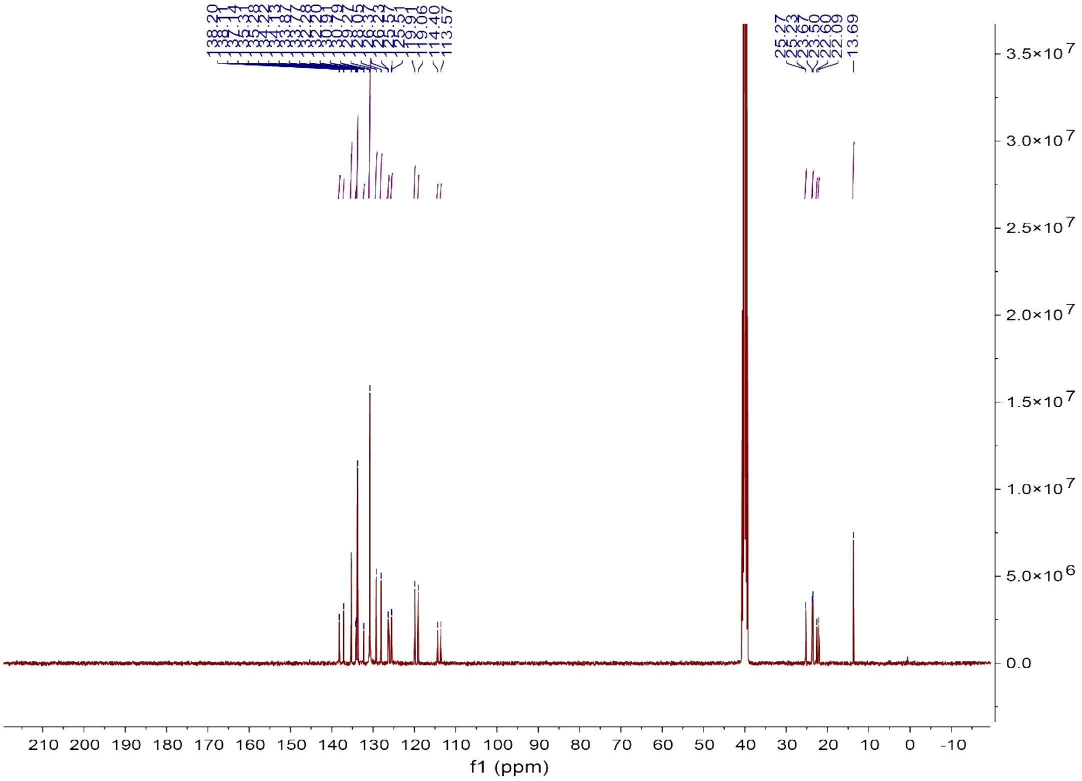

Fig. S44. ^{13}C NMR spectra of **NP-4C** in DMSO-d_6

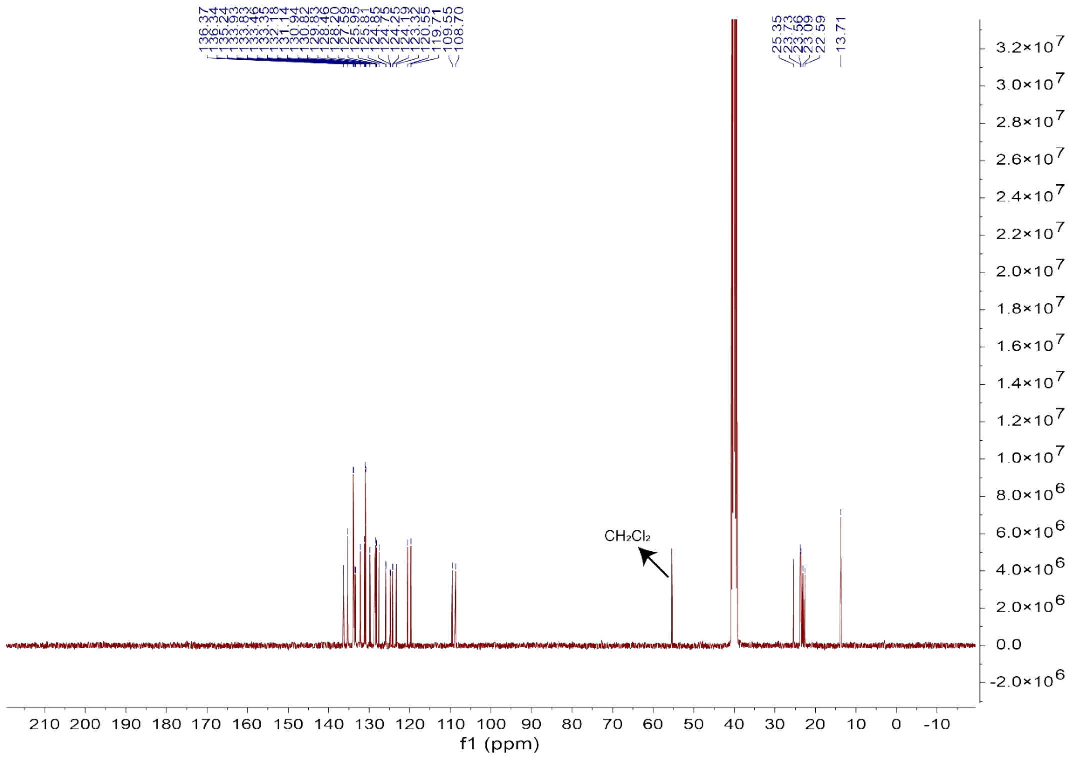

Fig. S45. ^{13}C NMR spectra of **PY-4C** in DMSO-d_6

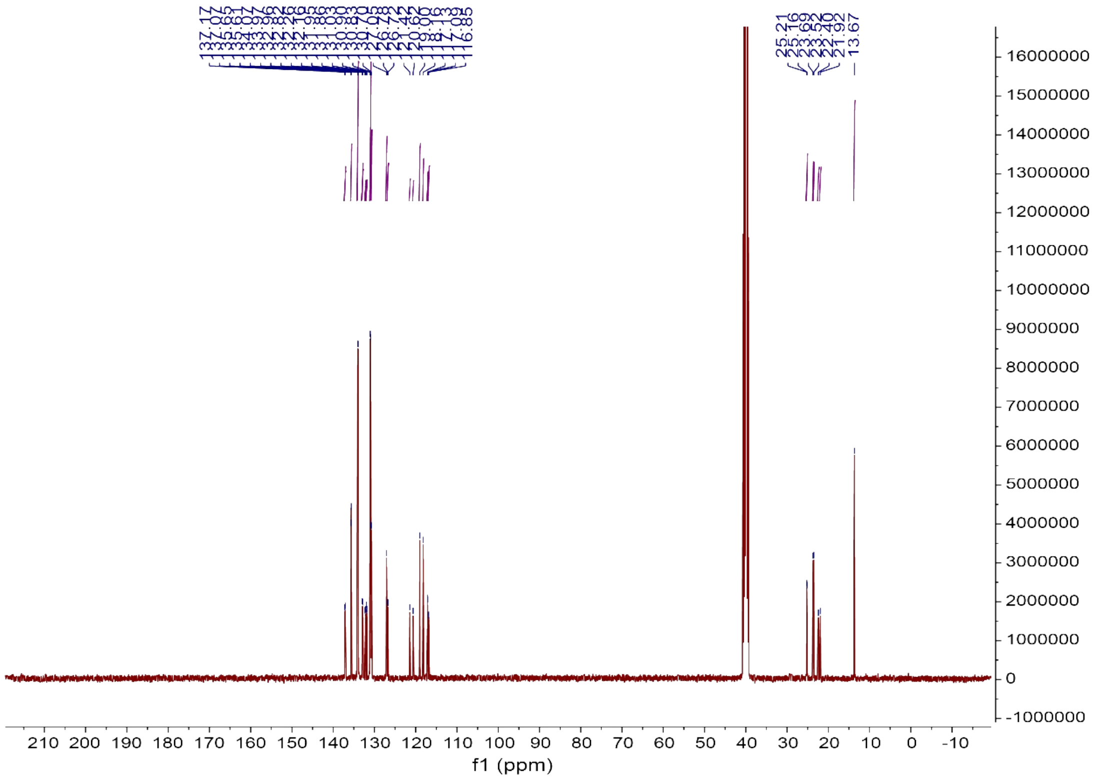

Fig. S46. ^{13}C NMR spectra of **CN-4C** in DMSO-d_6

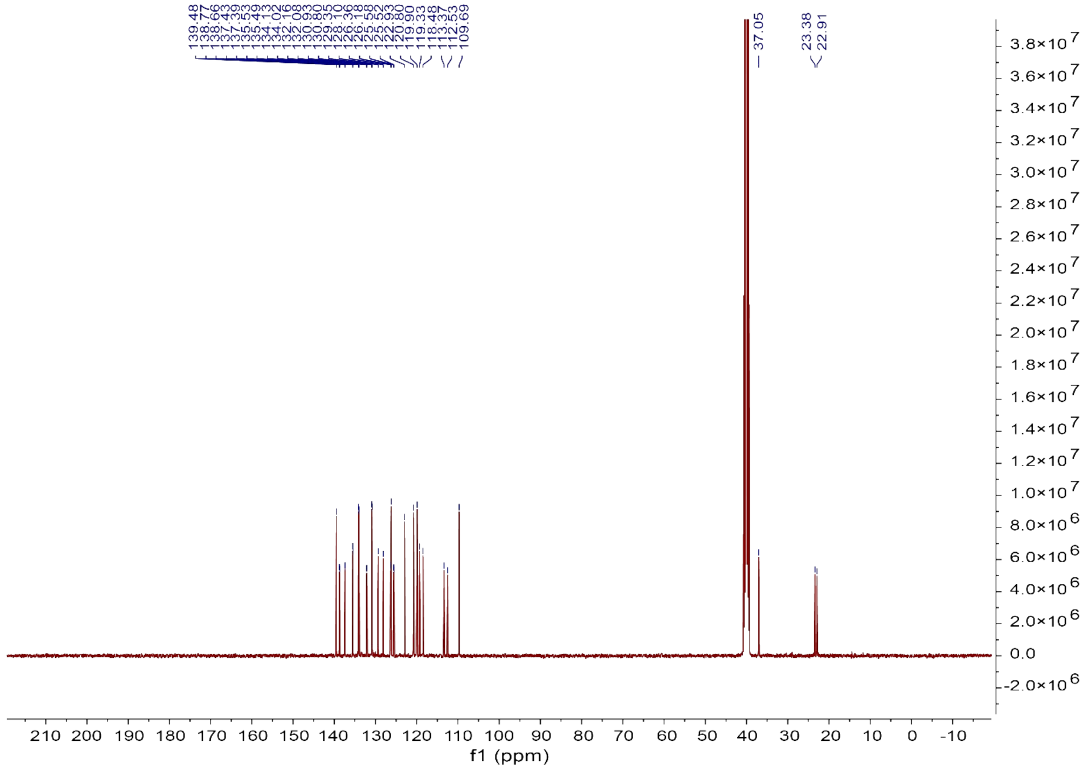

Fig. S47.^{13}C NMR spectra of **NP-2C-Cz** in DMSO-d_6

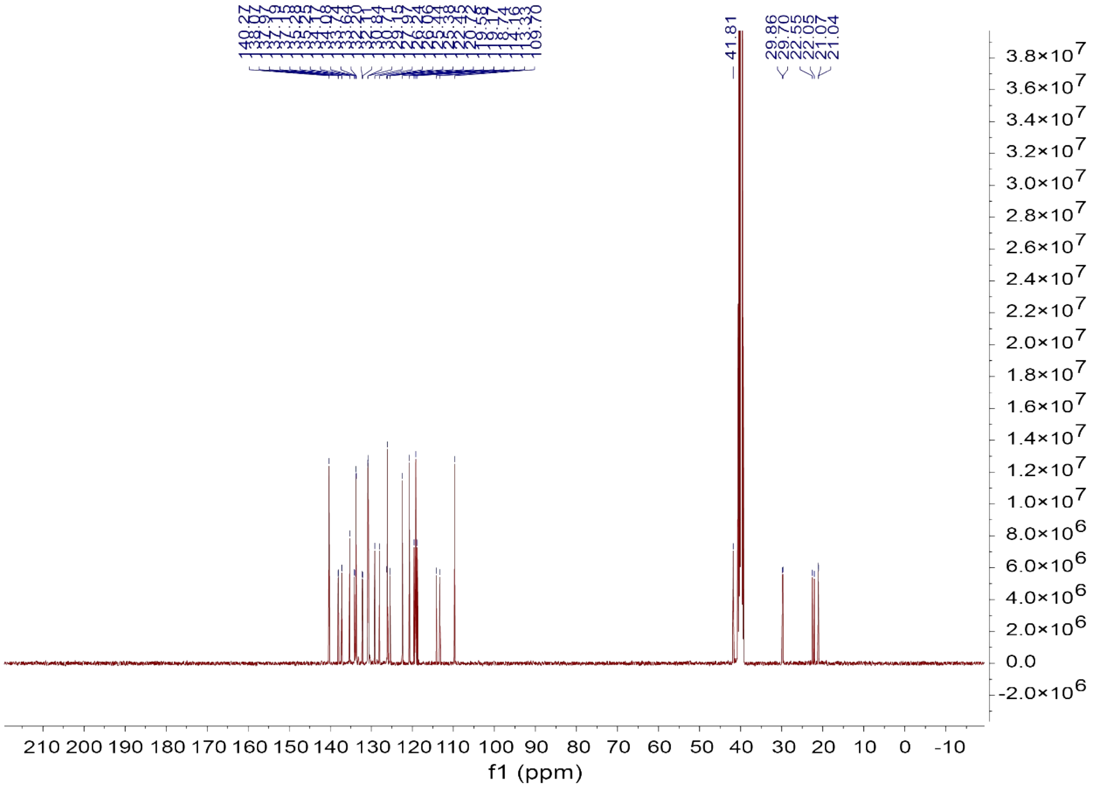

Fig. S48. ^{13}C NMR spectra of **NP-4C-Cz** in DMSO-d_6

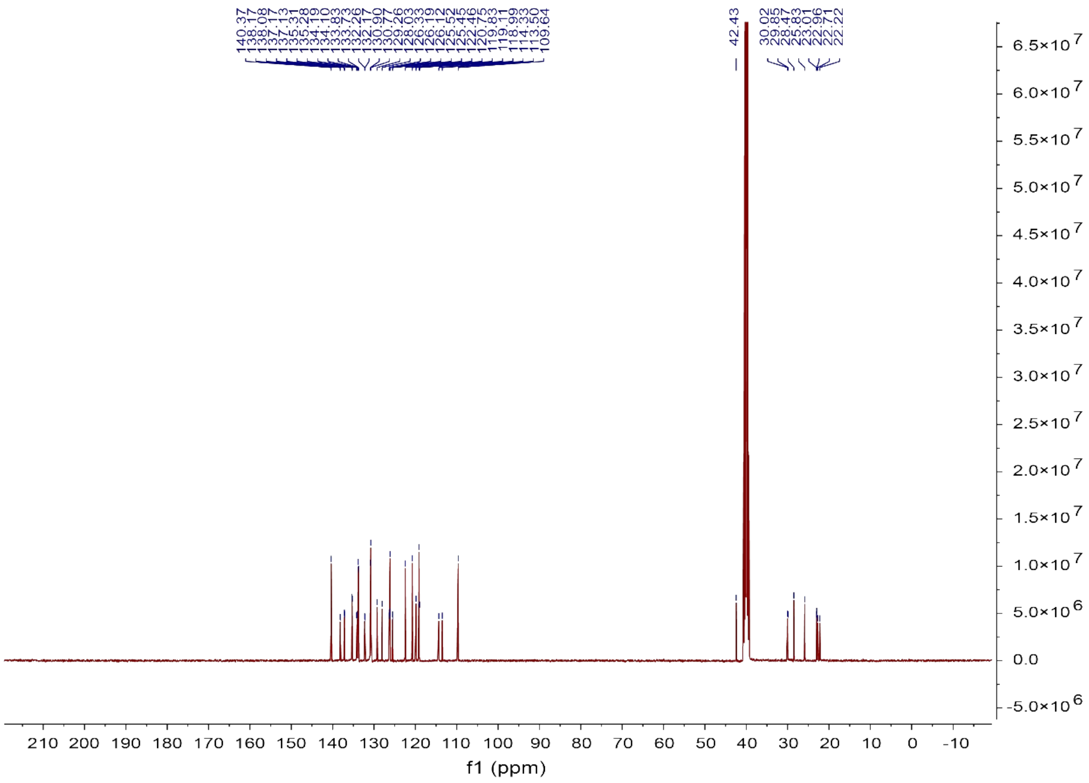

Fig. S49. ^{13}C NMR spectra of **NP-6C-Cz** in DMSO-d_6

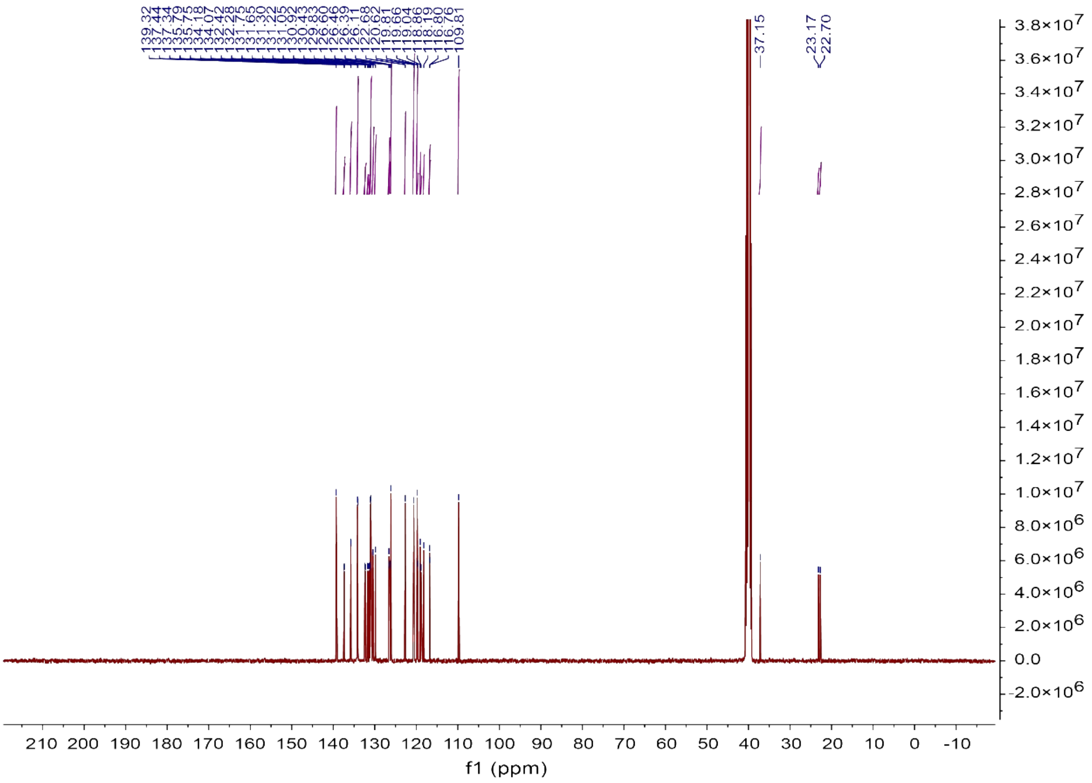

Fig. S50. ^{13}C NMR spectra of **CN-2C-Cz** in DMSO-d_6

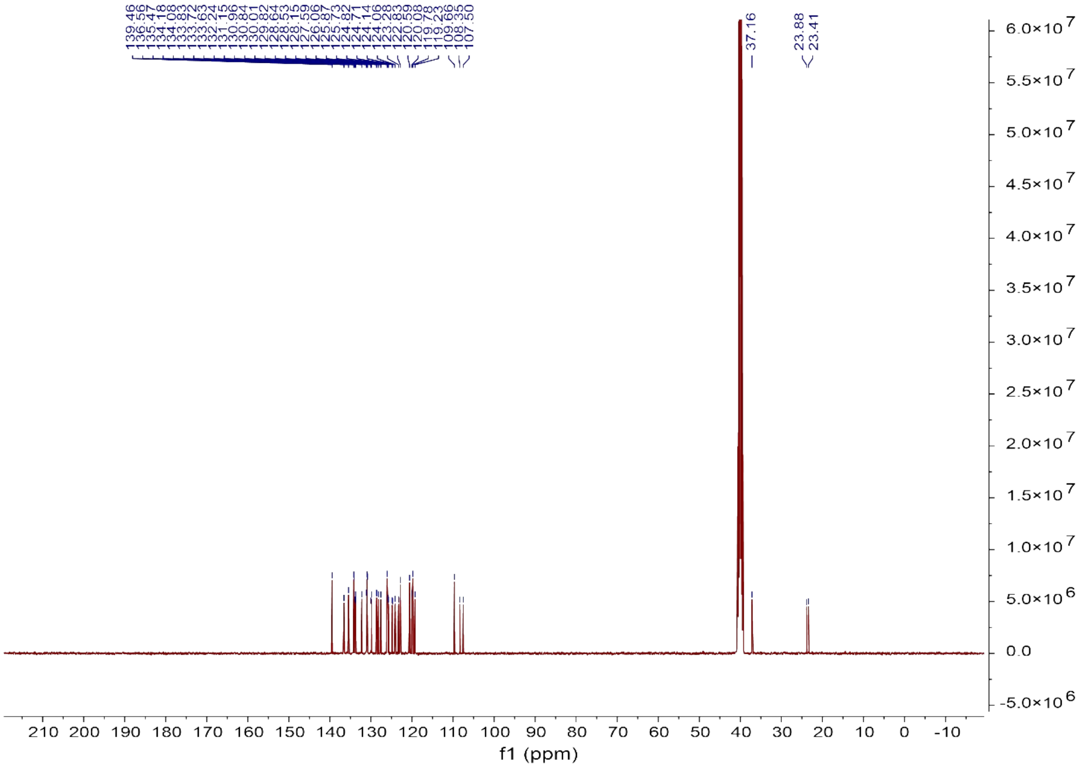

Fig. S51. ^{13}C NMR spectra of **PY-2C-Cz** in DMSO-d_6
